# Supplementary material for: Molecular bilayer graphene
Source: Nat Commun. 2019 Jul 11;10:3057. doi: 10.1038/s41467-019-11098-9 (PMC6624274; doi:10.1038/s41467-019-11098-9)
Supplement: Supplementary file 1 — Supplementary Information [file 41467_2019_11098_MOESM1_ESM.pdf]

Supplementary Information for Molecular Bilayer Graphene

Xin-Jing Zhao[1+], Hao Hou[1+], Xue-Ting Fan[1+], Yu Wang[1], Yu-Min Liu[1], Chun Tang[1], Shun-He Liu[1], Peng-Peng Ding[1], Jun Cheng[1]*, Dong-Hai Lin[1], Cheng Wang[1], Ye Yang[1], Yuan-Zhi Tan[1]*

[1] Collaborative Innovation Center of Chemistry for Energy Materials, State Key Laboratory for Physical Chemistry of Solid Surfaces, and Department of Chemistry, College of Chemistry and Chemical Engineering, Xiamen University, Xiamen 361005
+These authors contributed equally to this work
*Corresponding author.
Email: yuanzhi_tan@xmu.edu.cn (T.Y.-Z.); chengjun@xmu.edu.cn (C.J.)

Supplementary Figures

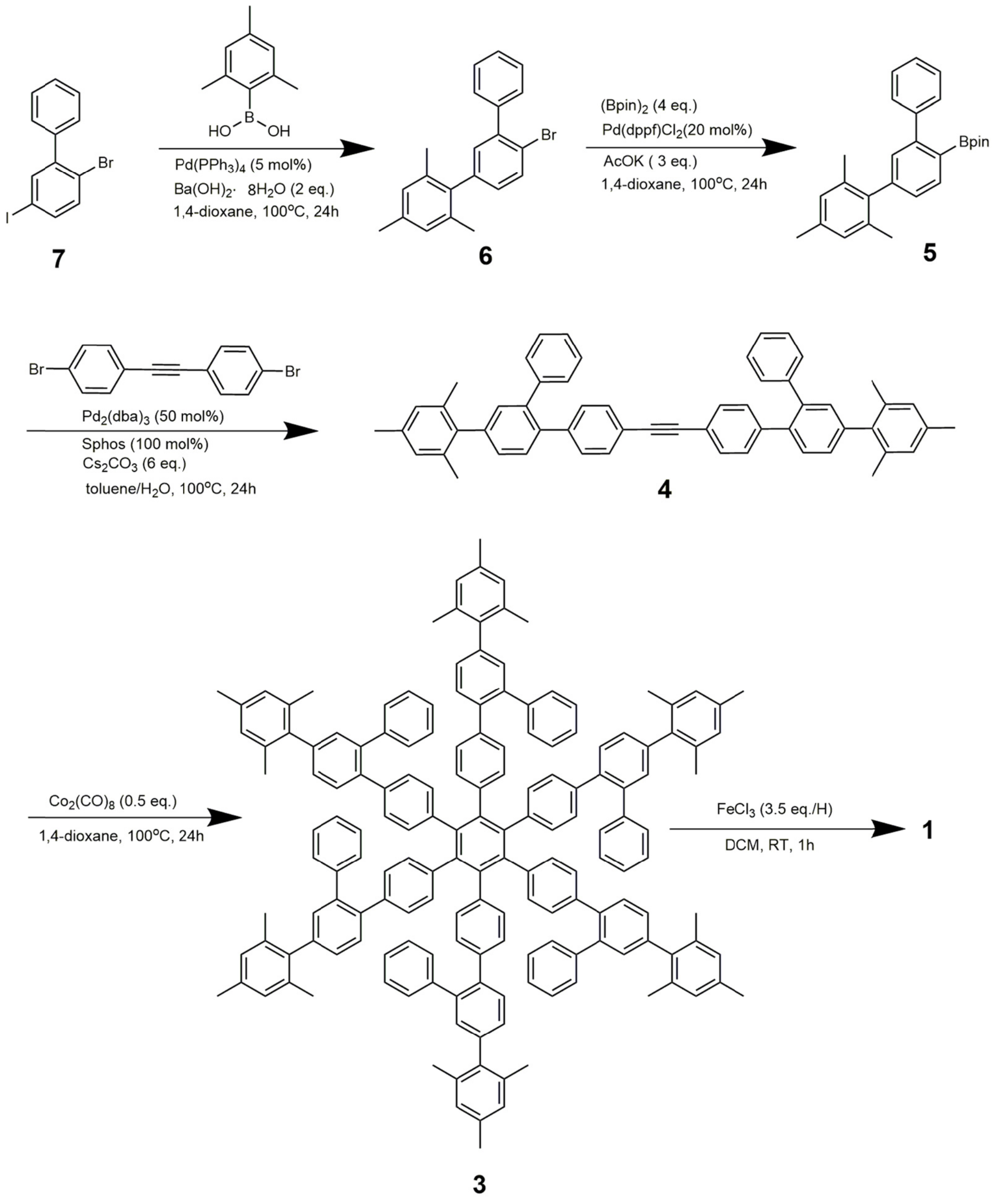

Supplementary Figure 1. Synthetic route of 1.

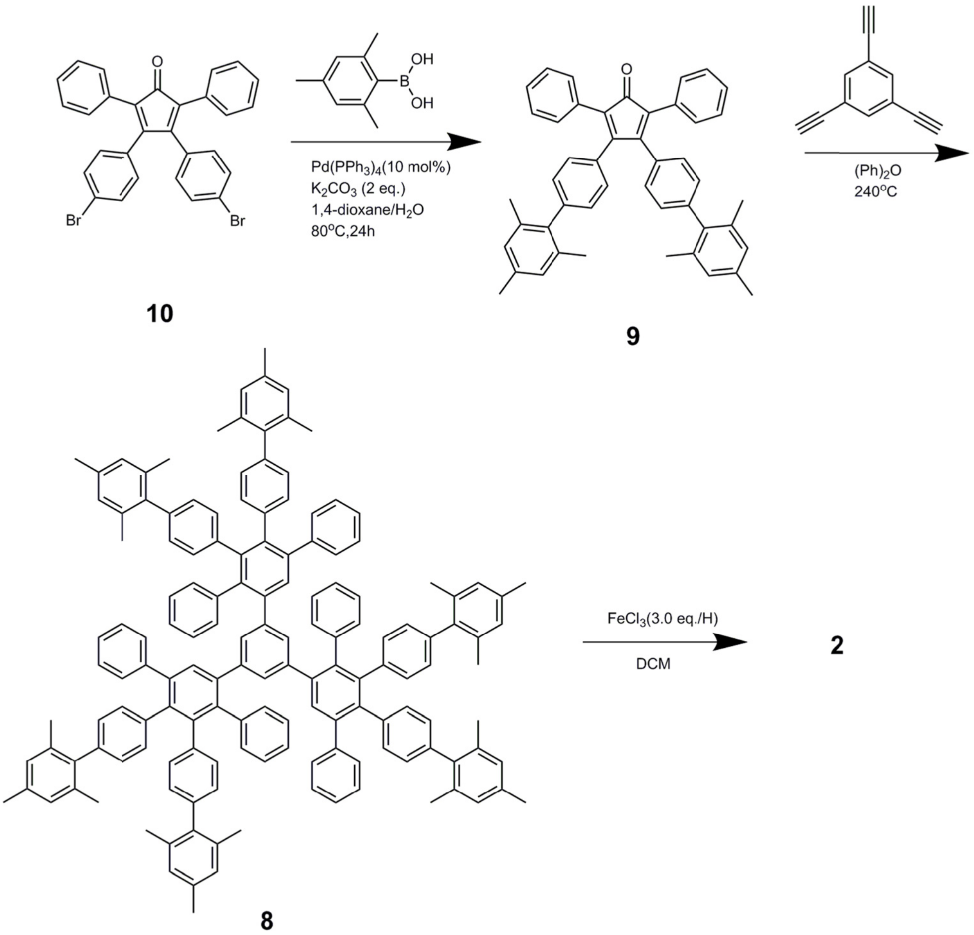

Supplementary Figure 2. Synthetic route of 2.

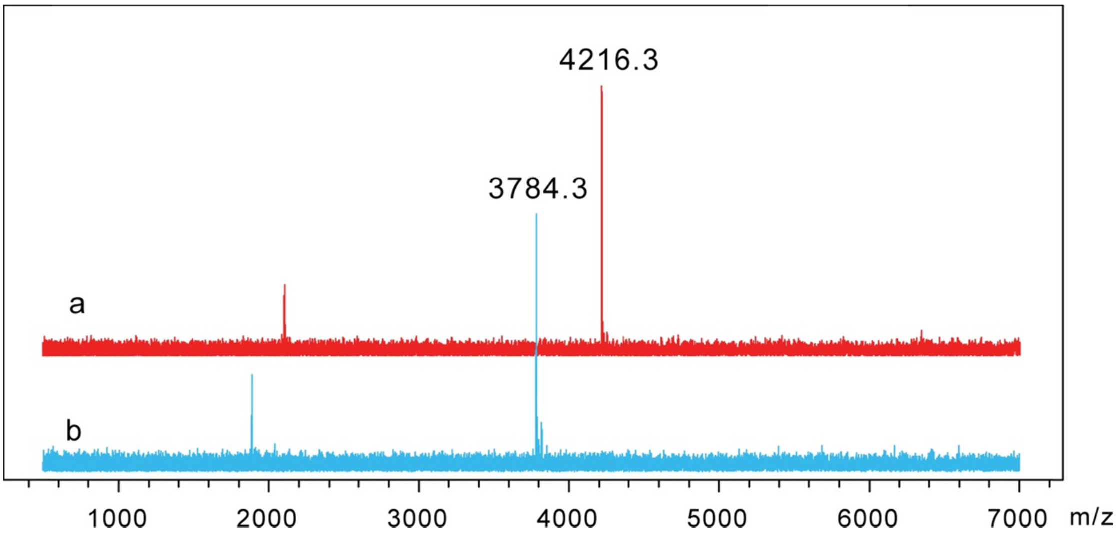

Supplementary Figure 3. Mass spectra of 1 (a) and 2 (b).

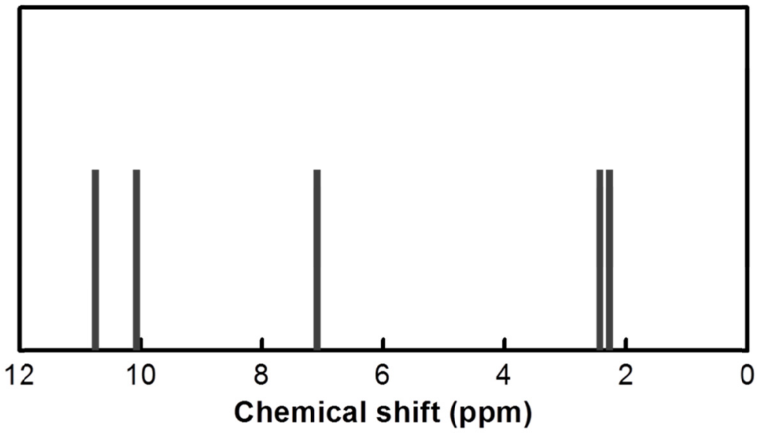

Supplementary Figure 4. Theoretically calculated ^{1}H NMR spectrum of the monolayer of MBLG 1. There are one peak for the aromatic hydrogen at the benzene ring and two peaks for methyl at the mesityl groups, due to the mirror symmetry along the graphene plane for the monolayer.

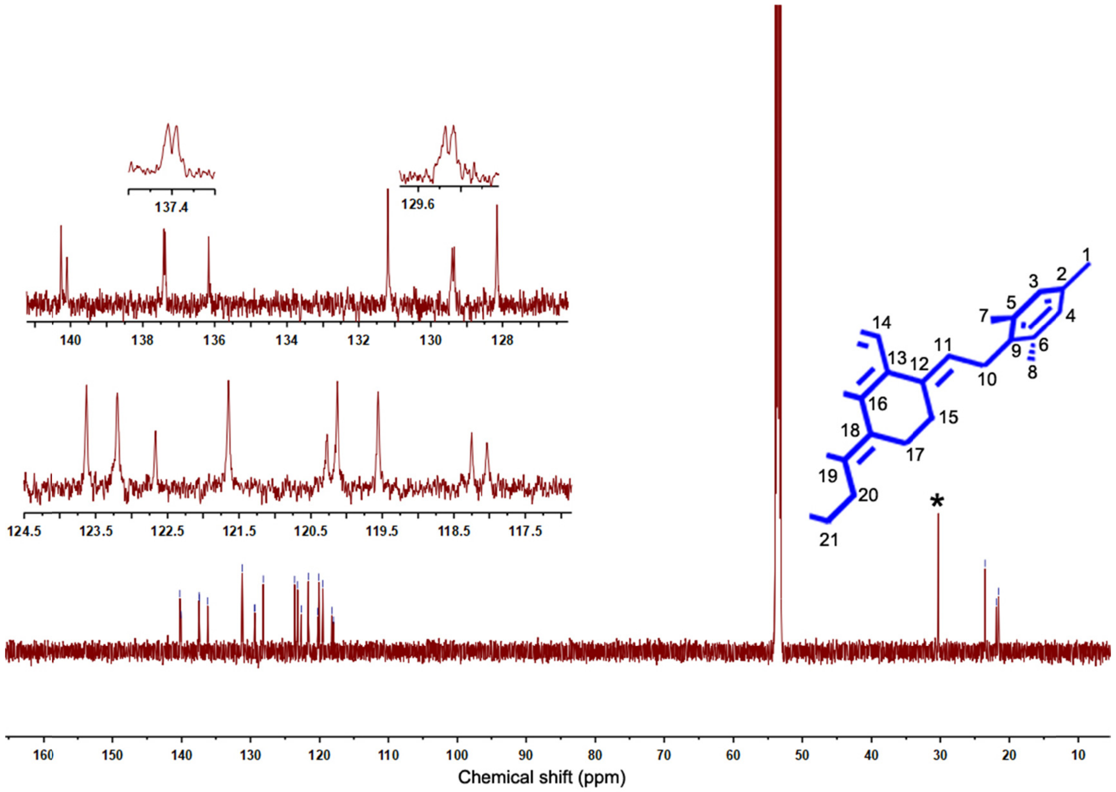

Supplementary Figure 5. ^{13}C NMR spectrum of 1 in CD_2Cl_2/CS_2. There are 18 peaks in the aromatic range (12 peaks of C_{114} core and 6 peaks of mesityl group) and 3 peaks in the alkyl range, consistent with the number of carbon species in the asymmetric unit of **1** (inserted figure). We could see each carbon atom of mesityl group is chemically inequivalent, which clearly demonstrates the bilayer structure of **1**. The peaks at 29.5 ppm is assigned to the signal of hexane, marked by asterisk. ^{13}C NMR (150 MHz, $CDCl_3$). δ = 140.26, 140.10, 137.42, 137.38, 136.17, 131.19, 129.41, 129.35, 128.15, 123.63, 123.20, 122.67, 121.65, 120.27, 120.13, 119.56, 118.25, 118.02, 23.53, 21.88, 21.57 ppm.

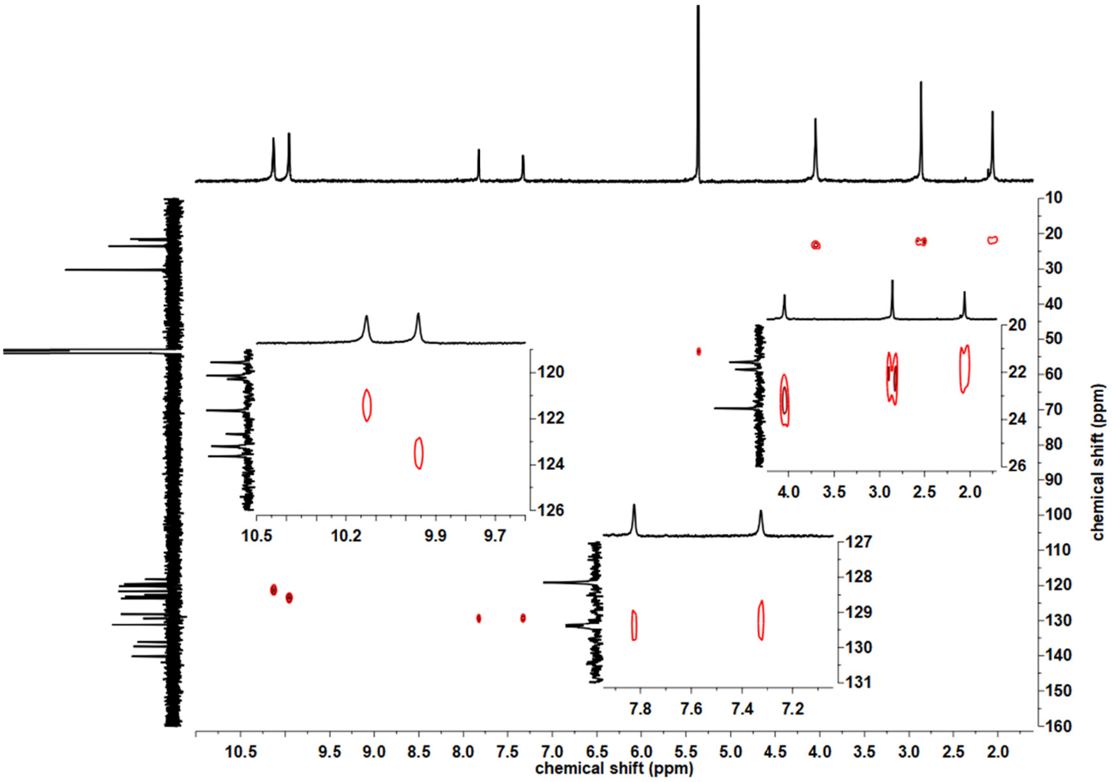

Supplementary Figure 6. ^{13}C-^{1}H COSY of 1 in CD_2Cl_2/CS_2. As shown, three methyl groups of mesityl are inequivalent.

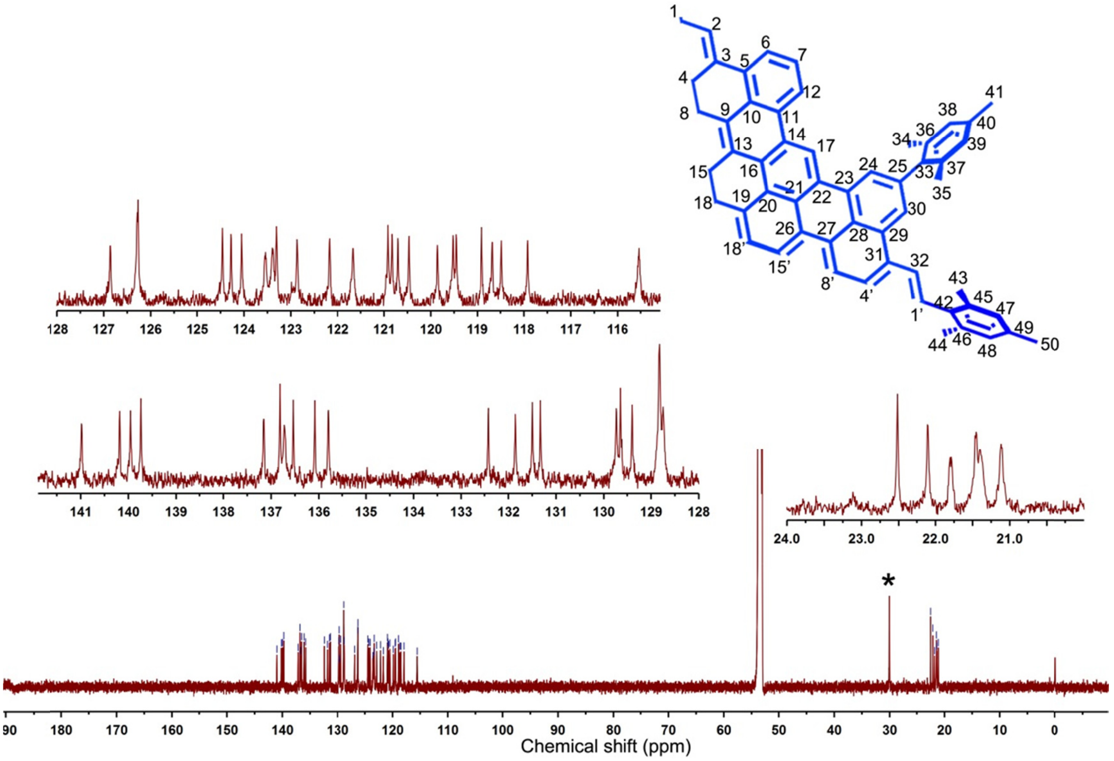

Supplementary Figure 7. ^{13}C NMR spectrum of 2 in CD_2Cl_2/CS_2. There are 44 peaks (32 peaks of C_{96} core and 12 peaks of two inequivalent mesityl groups) in the aromatic range and 6 peaks in the alkyl range, consistent with the number of carbon species in the asymmetric unit of **2** (inserted figure). We could see each carbon atom of mesityl groups is inequivalent. The peaks at 29.5 ppm is assigned to the signal of hexane, marked by asterisk. ^{13}C NMR (150 MHz, $CDCl_3$) δ = 140.98, 140.18, 139.96, 139.74, 137.15, 136.81, 136.72, 136.54, 136.08, 135.80, 132.42, 131.85, 131.50, 131.33, 129.74, 129.65, 129.62, 129.40, 128.82, 128.75, 126.86, 126.28, 126.27, 124.46, 124.28, 124.05, 123.56, 123.38, 123.31, 122.87, 122.17, 121.66, 120.92, 120.83, 120.70, 120.47, 119.86, 119.52, 119.46, 118.90, 118.67, 118.48, 117.92, 115.54, 22.52, 22.11, 21.79, 21.45, 21.40, 21.12 ppm.

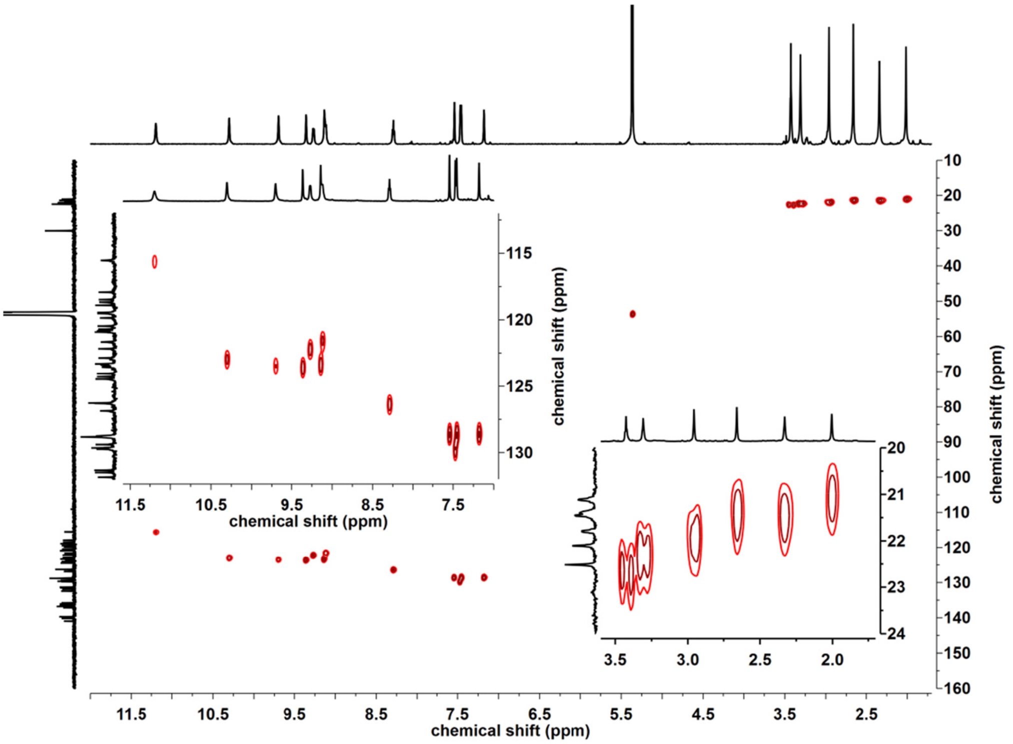

Supplementary Figure 8. ^{13}C-^{1}H COSY of 2 in CD_2Cl_2/CS_2. As shown, six methyl groups of mesityl are inequivalent.

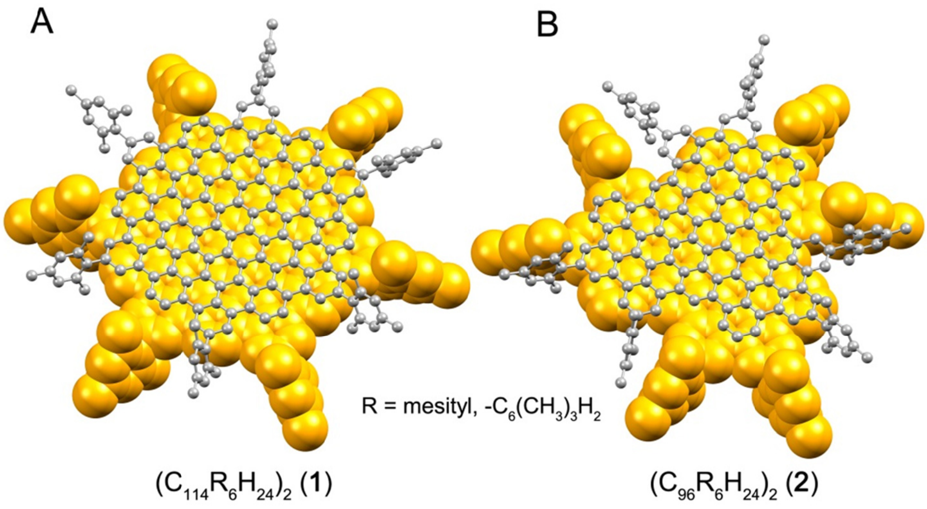

Supplementary Figure 9. Optimized structure of 1 (A) and 2 (B) by DFT calculations. The structures are optimized by density functional theory (DFT) calculation. One layer of MBLGs is illustrated with the spacefill model and the other layer with the ball-and-stick model. All hydrogens are omitted for clarity.

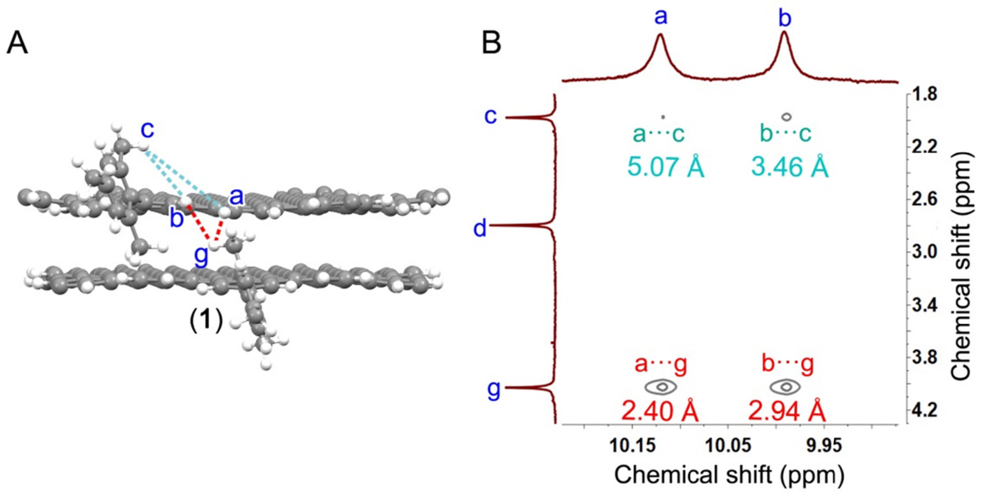

Supplementary Figure 10. H···H proximity in 1. A. typical H···H proximity found in **1**. The interlayer and intralayer H···H proximity are represented as red and cyan dashed lines, respectively. The rest mesityl groups are omitted for clarity. B. expanded 2D NOESY of **1**, showing interlayer protons coupling. The detailed **H**···H distances are labeled in B.

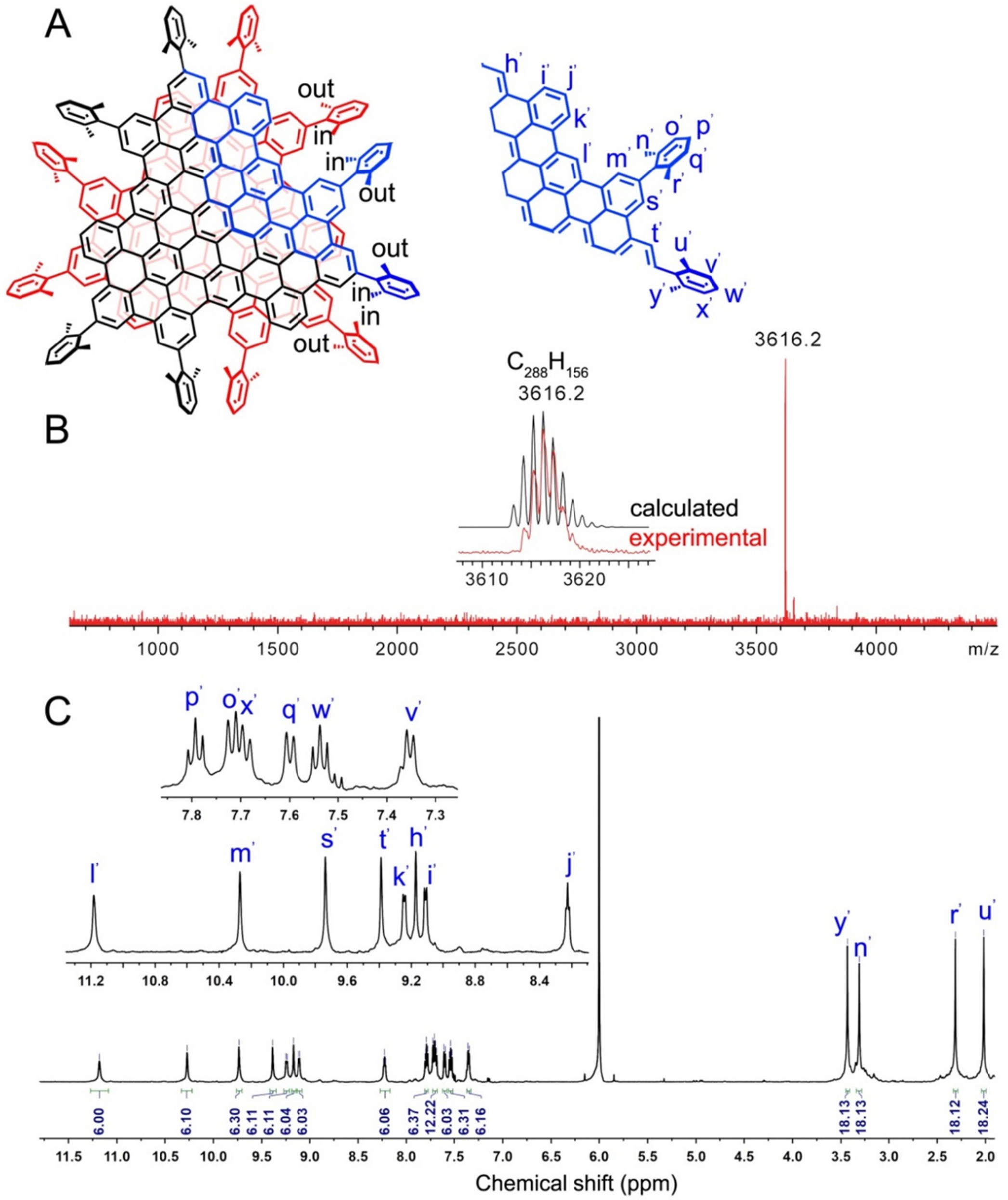

Supplementary Figure 11. Structure (A), mass spectrum (B) and ^{1}H NMR spectrum (C) of 2'. 2' = $[C_{96}H_{24}(C_6H_3(CH_3)_2)_6]_2$ The synthetic procedure of **2'** is similar to that of **2** by changing 2,4,6-trimethylphenylboronic acid into 2,6-dimethylphenylboronic acid. All of the proton resonances are assigned with the assistance of 2D NOESY (see supplementary information and Supplementary Figure 33-35). ^{1}H NMR (600 MHz, $C_2D_2Cl_4$): δ = 11.18 (s, 6H), 10.27 (s, 6H), 9.74 (s, 6H), 9.39 (s, 6H), 9.24 (d, 6H), 9.17 (s, 6H), 9.11 (d, 6H), 8.24-8.21 (t, 6H), 7.81-7.78 (t, 6H), 7.73-7.68 (m, 12H), 7.60 (d, 6H), 7.55-7.52 (t, 6H), 7.35(d, 6H), 3.43 (s, 18H), 3.31 (s, 18H), 2.31 (s, 18H), 2.02 (s, 18H) ppm.

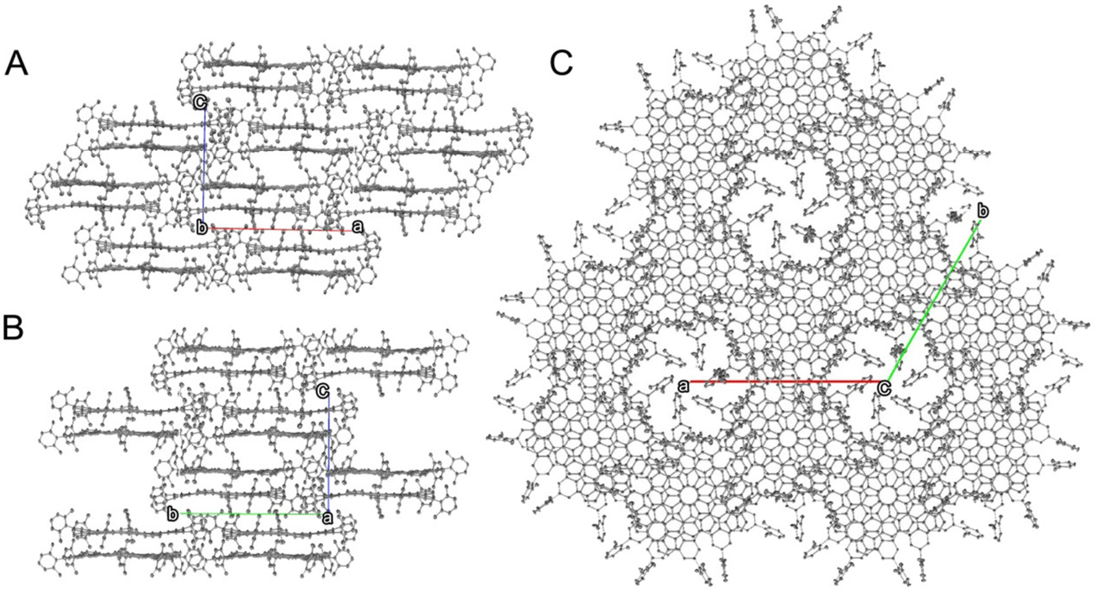

Supplementary Figure 12. Crystal packing of 2'. Views along the b (A), a (B) and c (C) axis. Co-crystalized solvent molecules (hexane and carbon disulfide) and hydrogens are omitted for clarity. The crystals of **2'** were grown by slowly diffusing n-hexane into the carbon disulfide solution of **2'**. Crystallographic data for **2'** has been deposited at the Cambridge Crystallographic Data Centre under deposition numbers CCDC 1873164. Crystal data: space group P-3 (no. 147), a = 27.1063(10) Å, c = 19.1800(10) Å, V = 12204.5(11) Å^3, Z = 2, T = 100.00(10) K, 34126 reflections measured (7.532° ≤ 2Θ ≤ 131.996°), 13797 unique (R_{int} = 0.0566, R_{sigma} = 0.0950) which were used in all calculations. The final R_1 was 0.0884 (I > 2σ(I)) and $wR_2$was 0.2801 (all data).

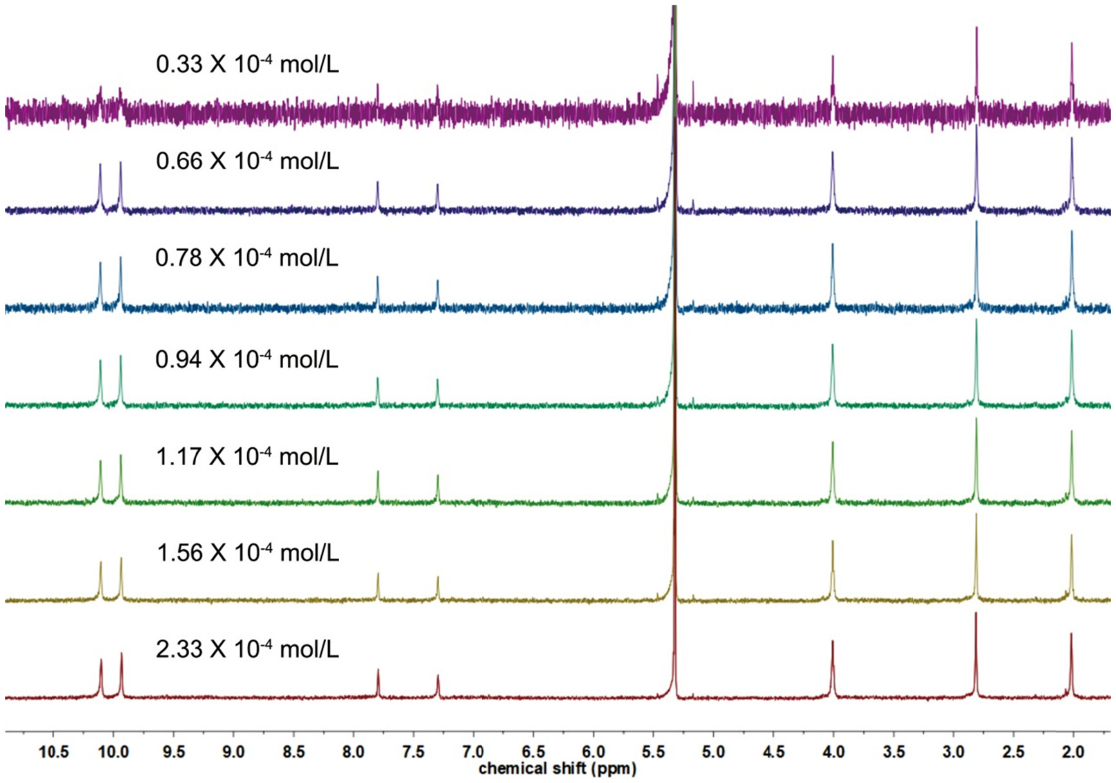

Supplementary Figure 13. ^{1}**H NMR spectra of 1 in** CD_2Cl_2/CS_2 **at different concentrations.**

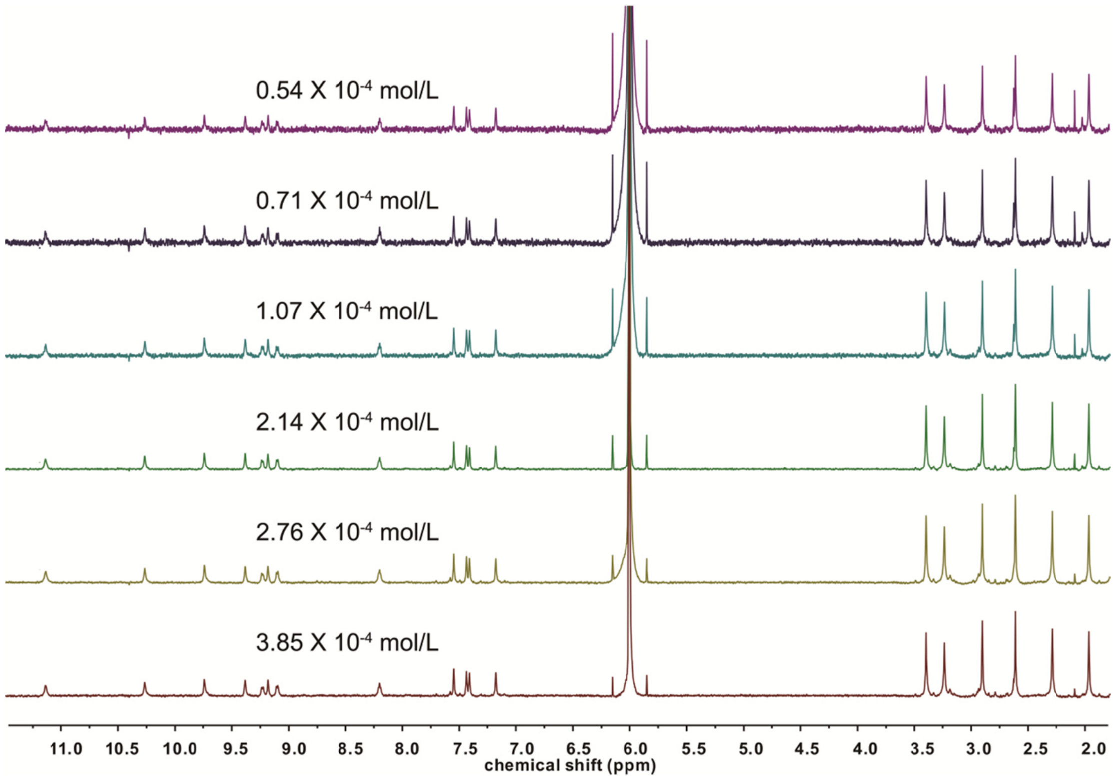

Supplementary Figure 14. ^{1}**H NMR spectra of 2 in** $C_2D_2Cl_4$ **at different concentrations.**

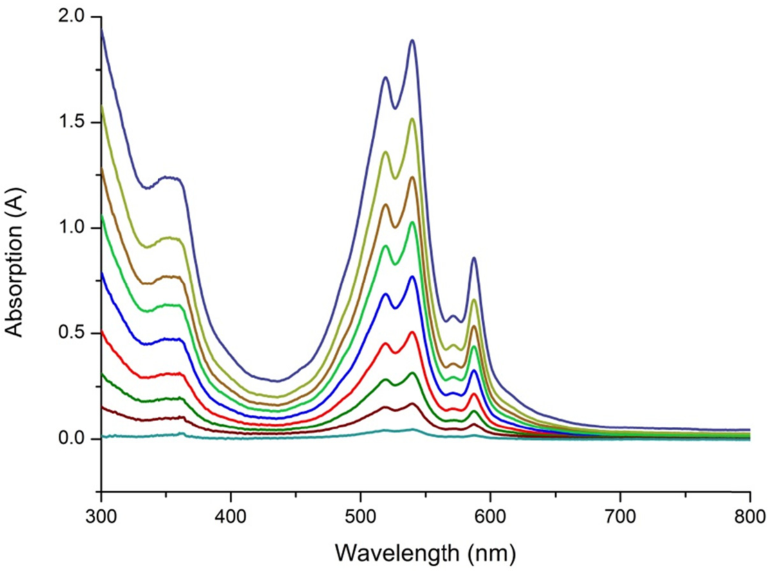

Supplementary Figure 15. UV-Vis spectra of 1 at the concentrations varying from 0.22×10^{-6} mol L^{-1} to 9.5×10^{-6} mol L^{-1} in toluene.

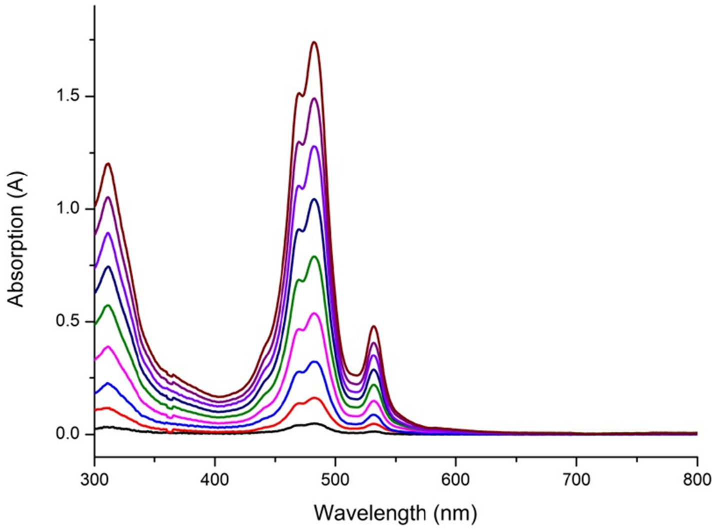

Supplementary Figure 16. UV-Vis spectra of 2 at the concentrations varying from 0.19×10^{-6} mol L^{-1} to 6.7×10^{-6} mol L^{-1} in toluene.

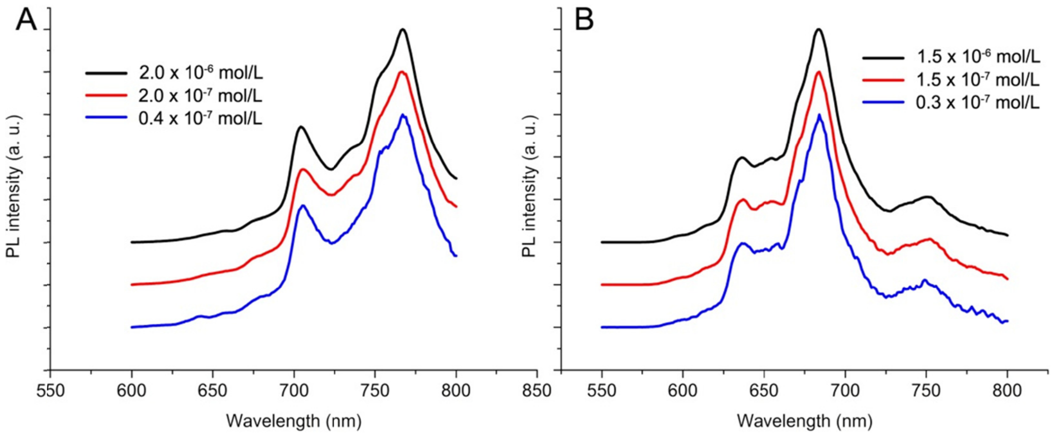

Supplementary Figure 17. Photo luminescence spectra of 1 (A) and 2 (B) at different concentrations. The intensity of photo luminescence at different concentration is normalized for clarity.

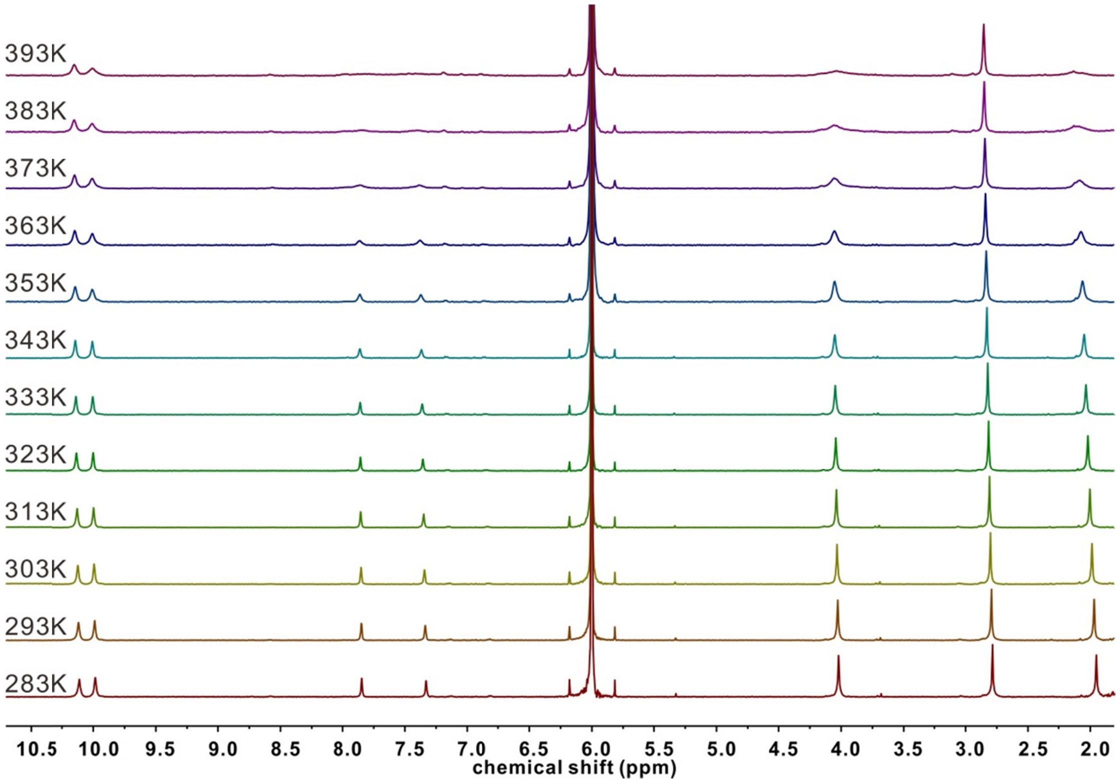

Supplementary Figure 18. ^{1}H NMR spectra of 1 in $C_2D_2Cl_4$ at different temperature.

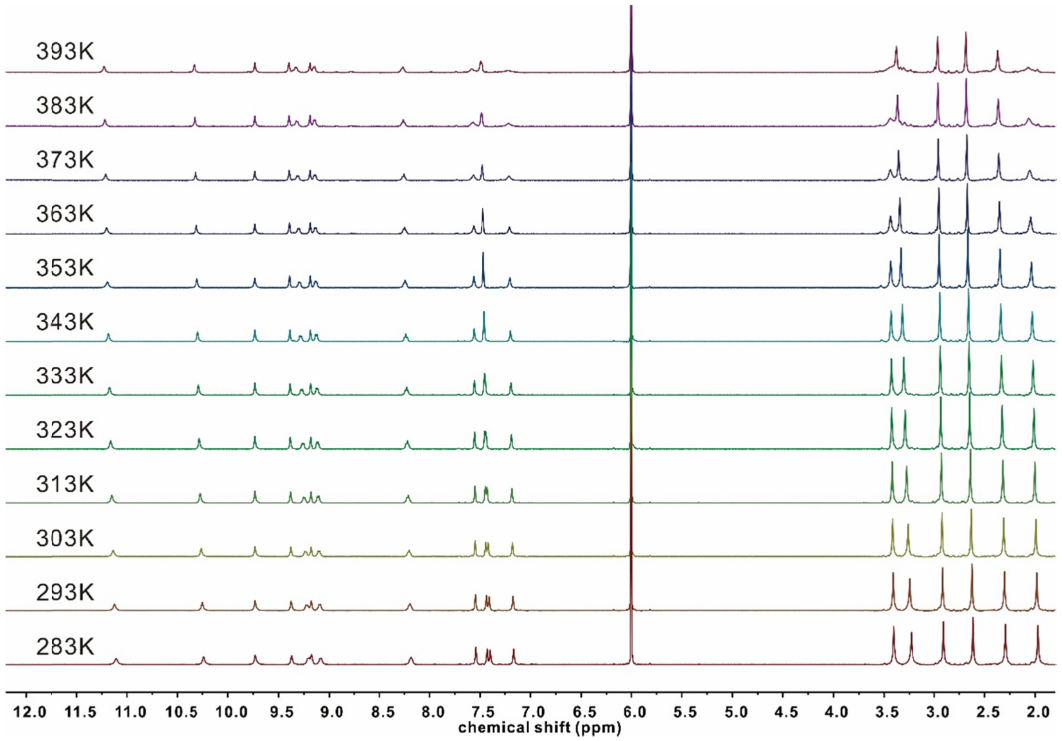

Supplementary Figure 19. ^1H NMR spectra of 2 in $C_2D_2Cl_4$ at different temperatures.

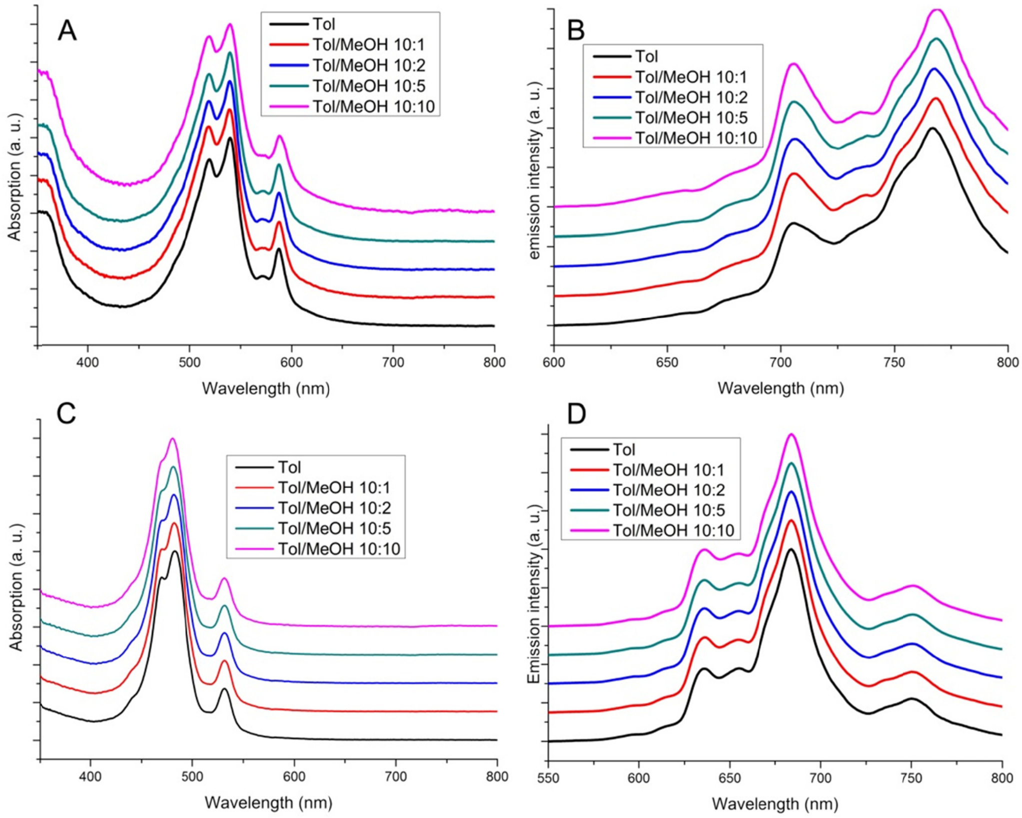

Supplementary Figure 20. Absorption and emission spectra of 1 (A, B) and 2 (C, D) in different solvents. Toluene = Tol, Methanol = MeOH.

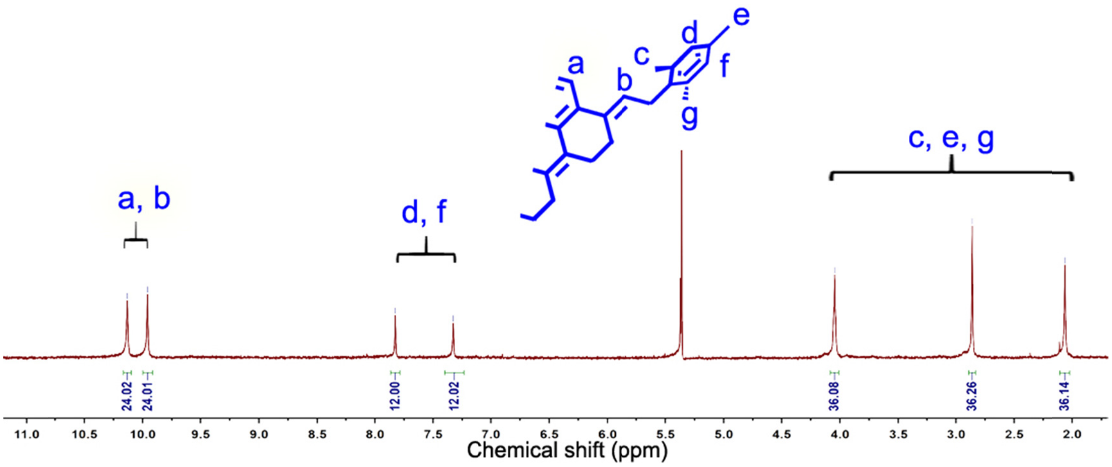

Supplementary Figure 21. ^{1}H NMR for 1. According to the chemical shifts, the signals are grouped into three categories. The insert is the asymmetric unit of 1. δ = 10.13 (s, 12H), 9.96 (s, 12H), 7.82 (s, 6H), 7.33 (s, 6H), 4.05 (s, 18H), 2.86 (s, 18H), 2.06 (s, 18H) ppm.

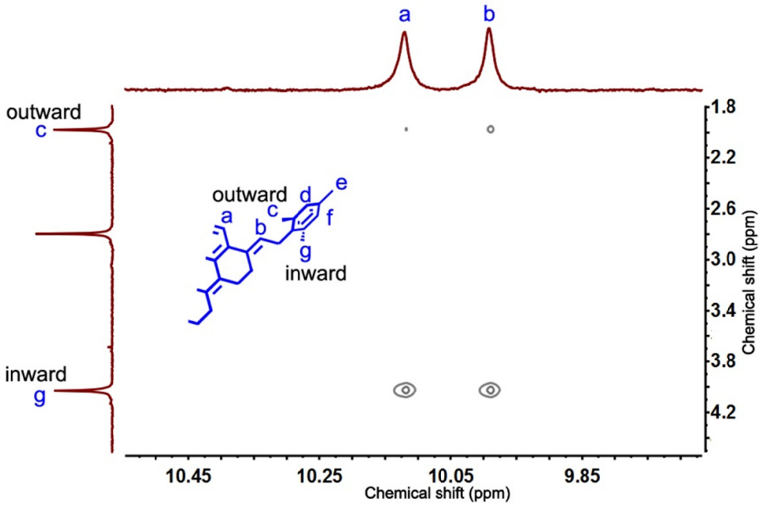

Supplementary Figure 22. Expanded 2D NOESY of 1 showing the proton coupling between protons (H_a and H_b) at C_{114} core and methyl of mesityl group (H_c, H_e and H_g). The insert is the asymmetric unit of **1**.

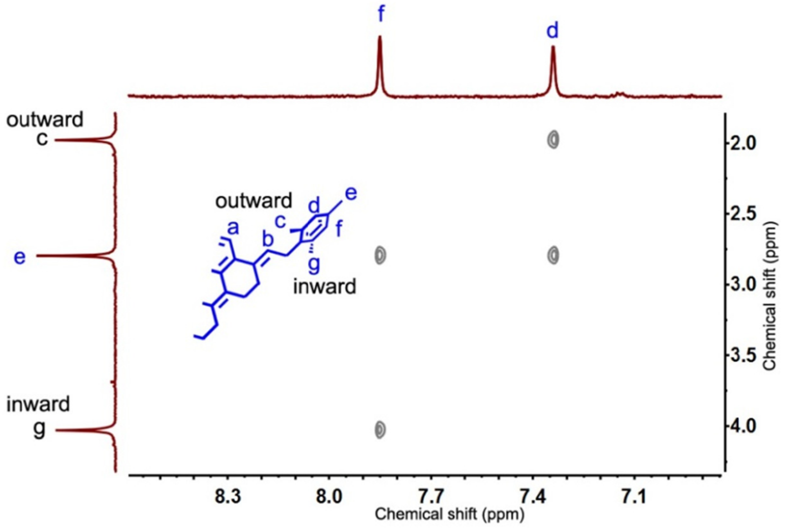

Supplementary Figure 23. Expanded 2D NOESY of 1 showing the proton coupling between protons (H_f and H_d) at benzene ring and those (H_c, H_g and H_e) at methyl of mesityl group. The insert is the asymmetric unit of **1**.

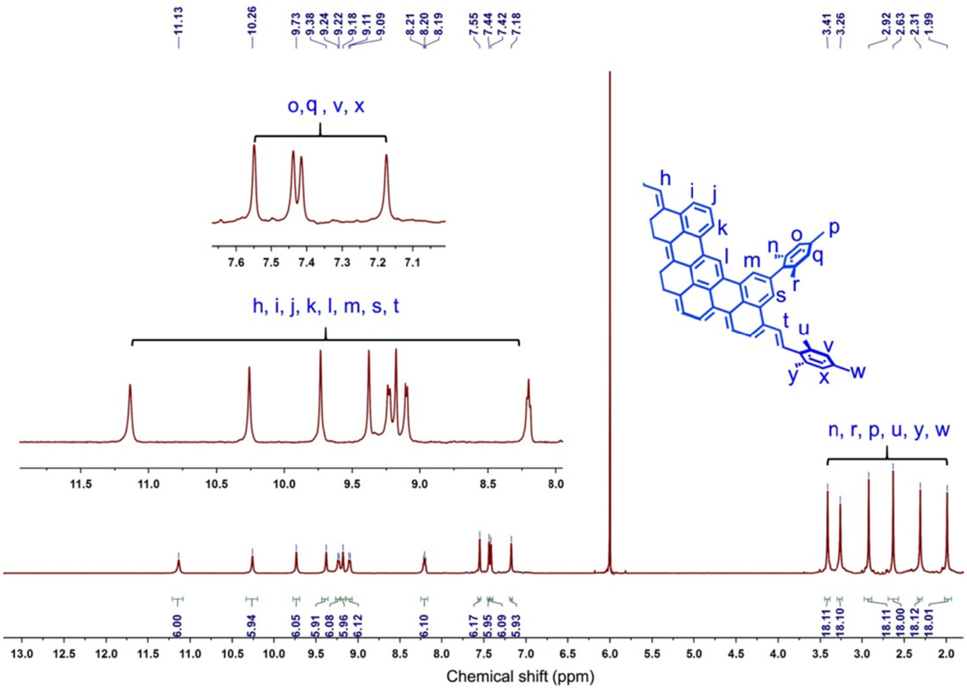

Supplementary Figure 24. ^{1}H NMR for 2. According to the chemical shifts, the signals are grouped into three categories. The insert is the asymmetric unit of **2**. δ= 11.13 (s, 6H), 10.26 (s, 6H), 9.73 (s, 6H), 9.38 (s,6H), 9.24 (d, 6H), 9.18 (s, 6H), 9.11 (d, 6H) 8.24-8.17 (t, 6H,), 7.55 (s, 6H), 7.44 (s, 6H), 7.42 (s, 6H), 7.18 (s, 6H), 3.41 (s, 18H), 3.26 (s, 18H), 2.92 (s, 18H), 2.63 (s, 18H), 2.31 (s, 18H), 1.99 (s, 18H) ppm.

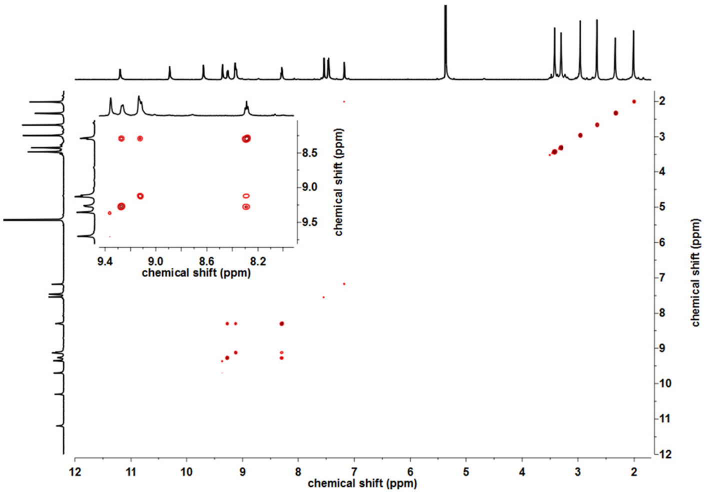

Supplementary Figure 25. ^1H-^1H COSY of 2 in CD_2Cl_2/CS_2

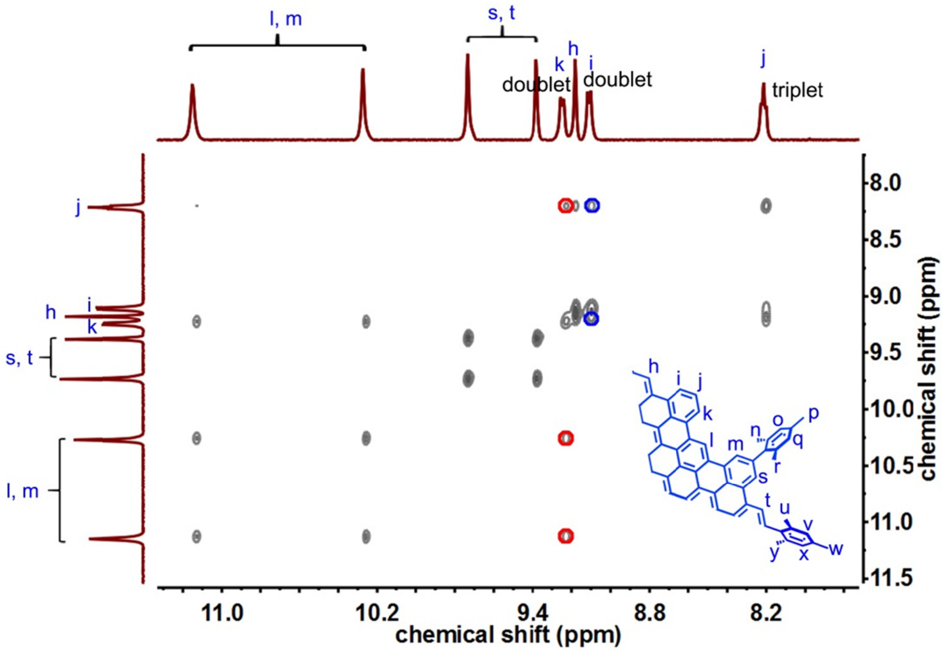

Supplementary Figure 26. Expanded 2D NOESY of 2 showing the proton coupling between protons at C_{96} core. The insert is the asymmetric unit of **2**. The red cycles represent the coupling of H_k to H_j, H_l, and H_m. The blue cycles represent the coupling of H_k to H_j and H_h

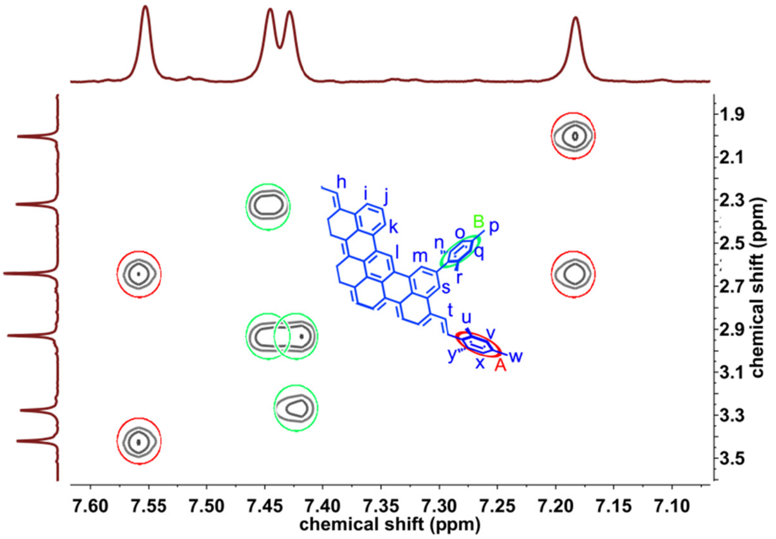

Supplementary Figure 27. Expanded 2D NOESY of 2 showing the proton coupling between protons at mesityl groups. The red cycles and blue cycles highlight the NOE signals from two inequivalent mesityl groups, respectively.

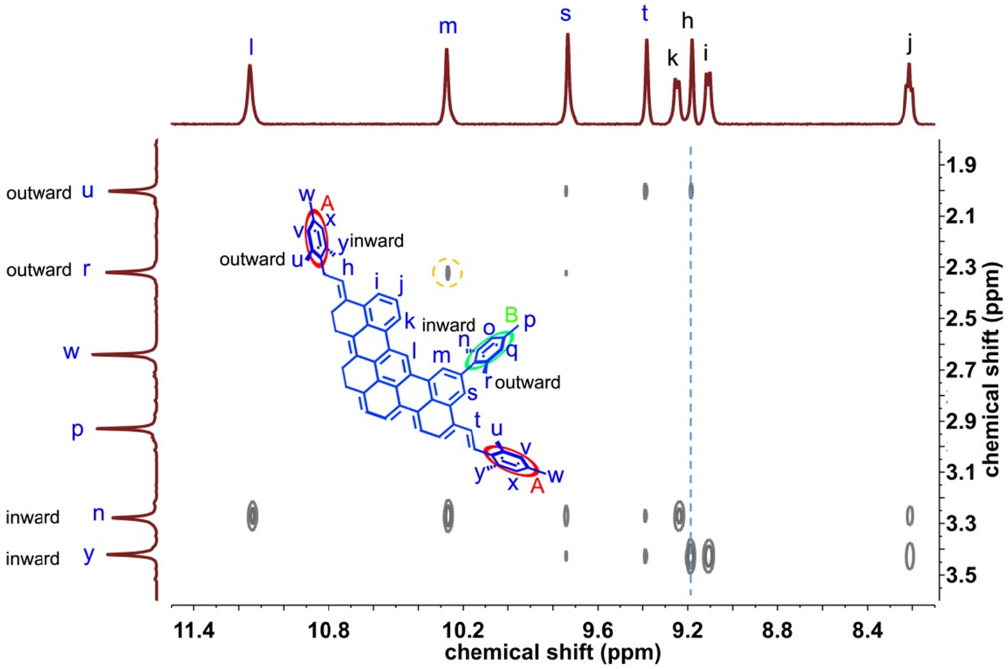

Supplementary Figure 28. Expanded 2D NOESY of 2 showing the proton coupling between protons at C_{96} core and protons at methyl of mesityl groups. The dashed blue line highlights the proton coupling of H_h to H_u and H_y. The dashed orange cycle highlights the proton coupling of H_r to H_m.

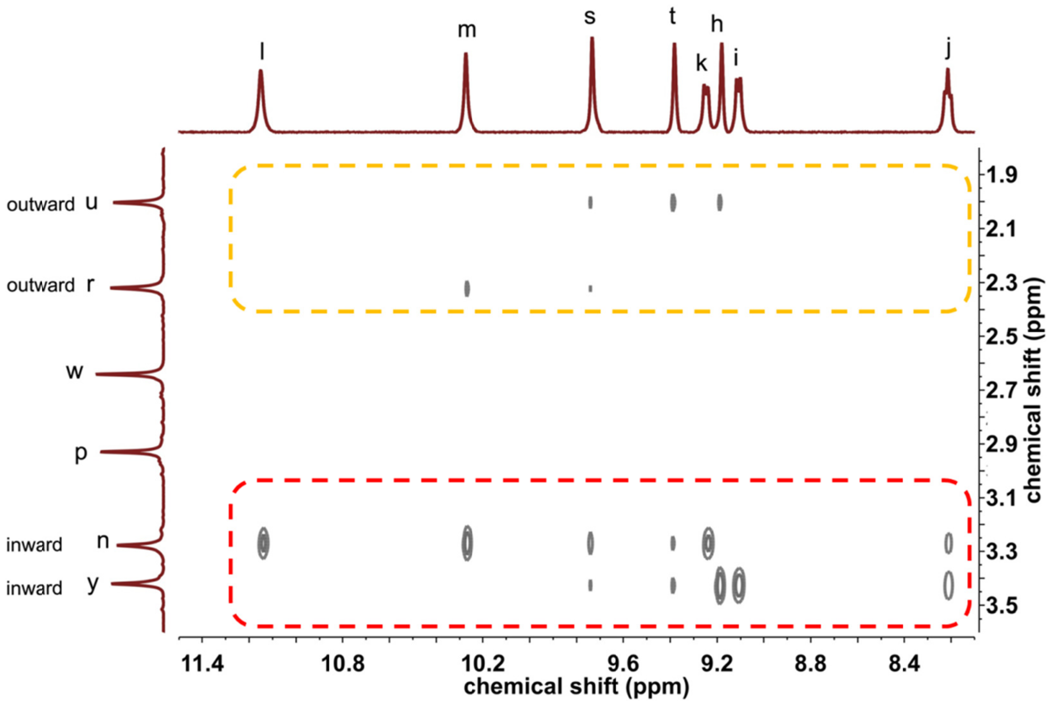

Supplementary Figure 29. Expanded 2D NOESY of 2 showing the proton coupling between protons at C_{96} core and those at methyl of mesityl groups. The orange and red dished rectangle highlight the intra- and interlayer proton-proton coupling, respectively.

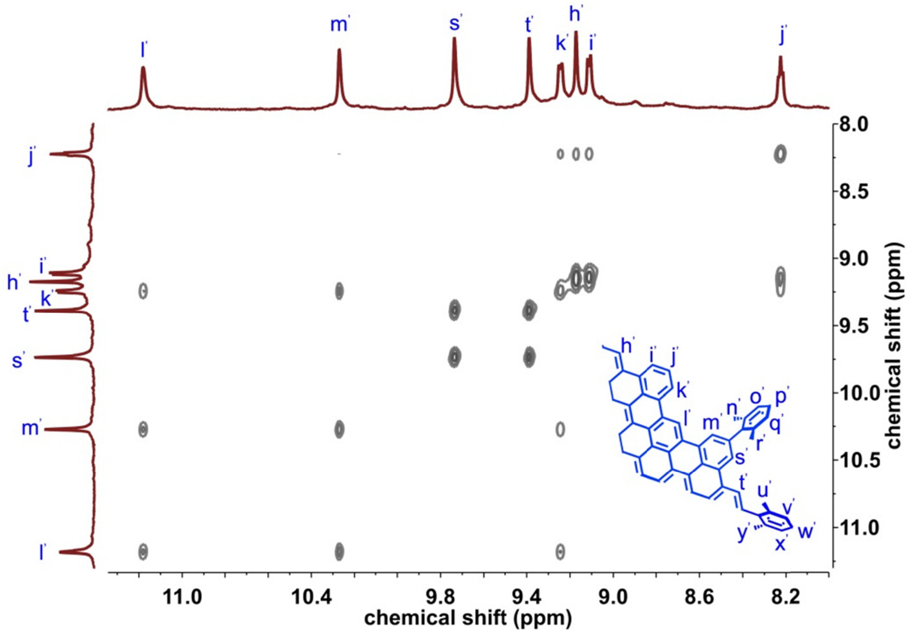

Supplementary Figure 30. Expanded 2D NOESY of 2' showing the proton coupling between protons at C_{96} core. The insert is the asymmetric unit of **2'**. Based on Supplementary Figure 30, $H_{j'}$, $H_{k'}$, $H_{i'}$ and $H_{h'}$ can be assigned.

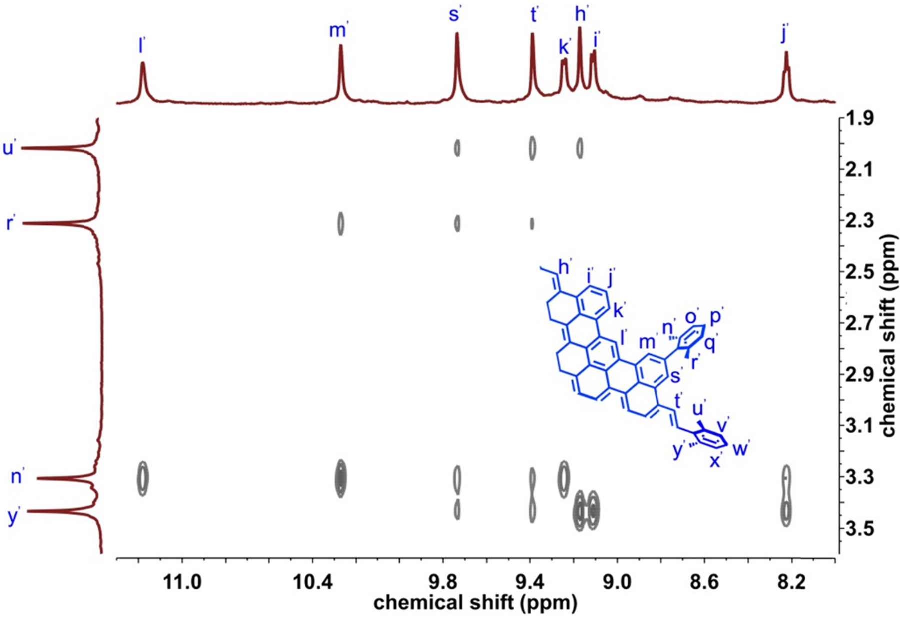

Supplementary Figure 31. Expanded 2D NOESY of 2' showing the proton coupling between protons at C_{96} core and protons at methyl of mesityl groups. Based on this NOESY, we can assign the peaks of $H_{n'}$, $H_{y'}$, $H_{r'}$, $H_{u'}$, $H_{l'}$, $H_{m'}$, $H_{s'}$ and $H_{t'}$, as we discussed for the NMR assignment of **2**.

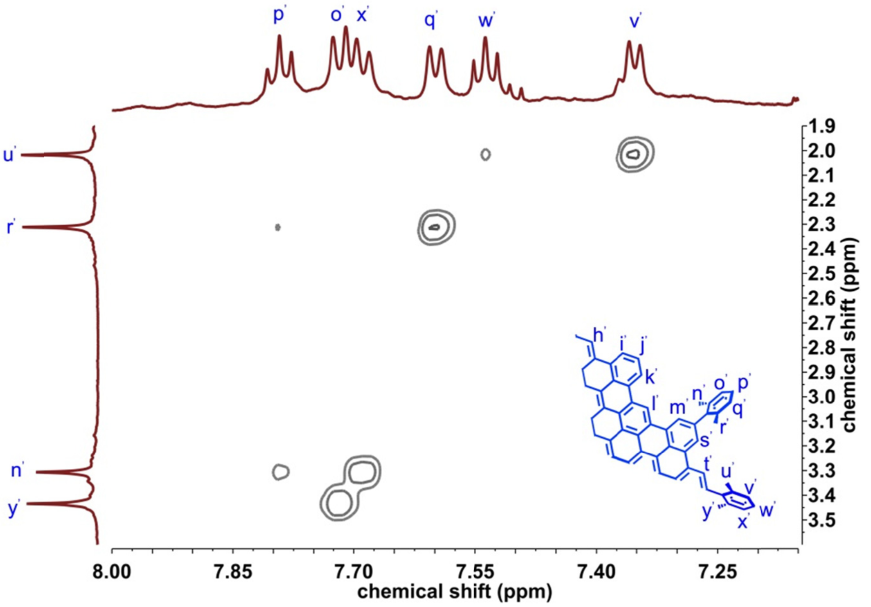

Supplementary Figure 32. Expanded 2D NOESY of 2' showing the proton coupling between protons at 2,6-dimethylphenyl groups. Based on this NOESY, the rest hydrogens ($H_{p'}$, $H_{o'}$, $H_{x'}$, $H_{q'}$, $H_{w'}$ and $H_{v'}$) are assigned.

Supplementary Note 1

Synthesis of compound **6**. Compound **7** is commercially available. A mixture of **7** (7.18 g, 20 mmol), 2,4,6-trimethylphenylboronic acid (3.94 g, 24 mmol), tetrakis(triphenylphosphine)palladium(0) [$Pd(PPh_3)_4$] (1.16 g, 1.0 mmol), and $Ba(OH)_2 \cdot 8H_2O$ (12.62 g, 40 mmol) in 1,4-dioxane (90 mL) and water (30 mL) was stirred under argon atmosphere at 100 °C for 24 h. After cooling to room temperature, hydrochloric acid (1.0 mol L^{-1}) was added and the mixture was extracted with CH_2Cl_2 (DCM) (100 mL×2). The organic layer was washed with water, dried with anhydrous $MgSO_4$, and concentrated under reduced pressure. The crude products were then purified by silica gel column chromatography (petroleum ether as eluent) to obtain **6** (3.80g, 54%) as a white solid. Mass found: m/z 350.1 [M^+]; Calcd. for $C_{21}H_{19}{}^{79}Br$ m/z 350.067. Elementary analysis. Found: C, 71.67%, H, 5.40% Calcd. for $C_{21}H_{19}Br$: C, 71.80%, H, 5.45%.

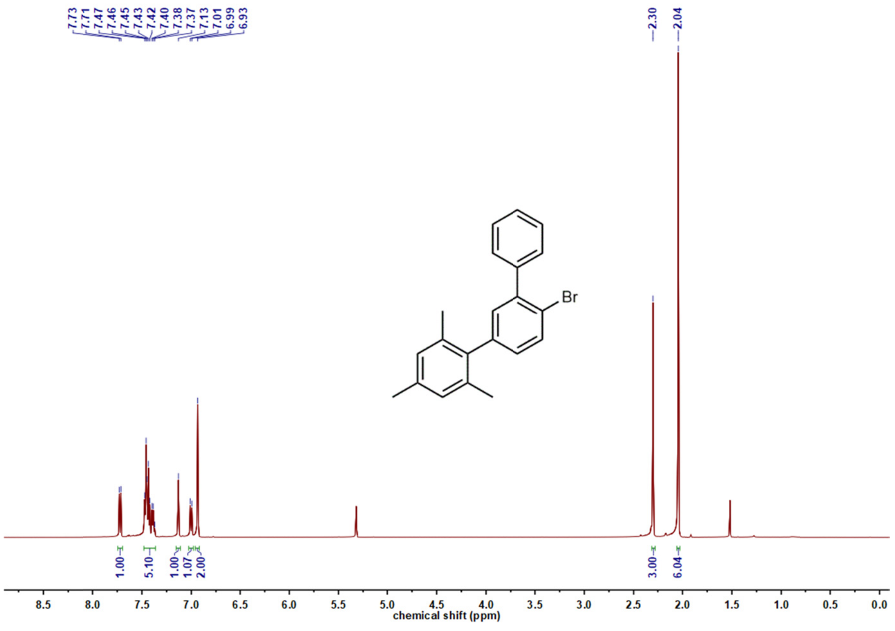

Supplementary Figure 33. ^{1}H NMR of compound 6. (600 MHz, CD_2Cl_2): δ = 7.72 (d, 1H), 7.47-7.37 (m, 5H), 7.13 (s, 1H), 7.00 (d, 1H), 6.93 (s, 2H), 2.30 (s, 3H), 2.04 (s, 6H) ppm.

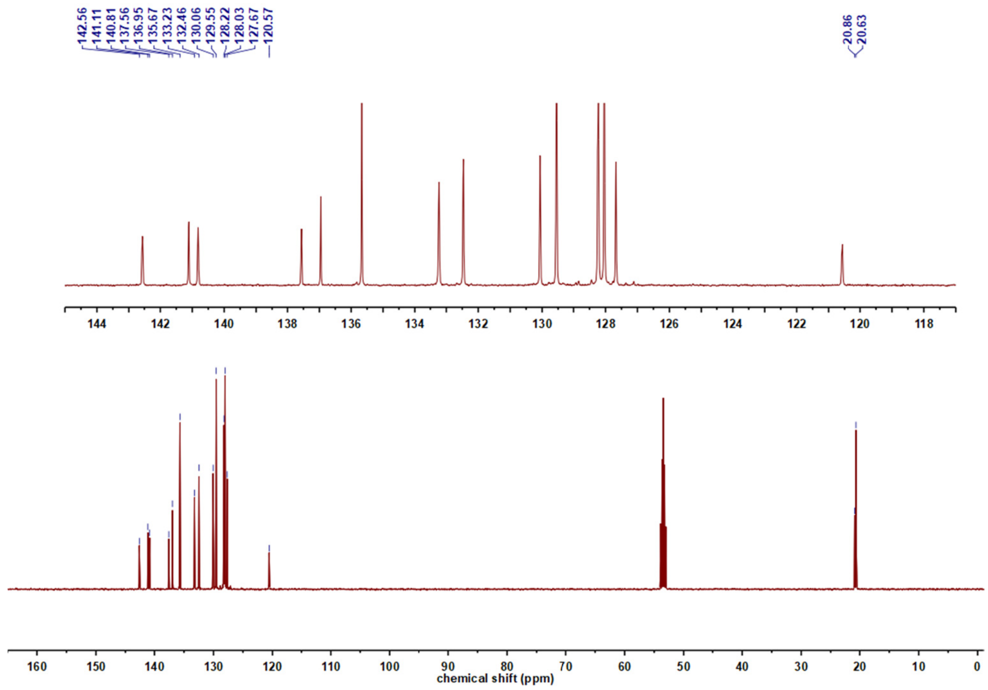

Supplementary Figure 34. ^{13}**C NMR of compound 6.** (150 MHz, CD_2Cl_2): δ = 142.56, 141.11, 140.81, 137.56, 136.95, 135.67, 133.23, 132.46, 130.06, 129.55, 128.22, 128.03, 127.67, 120.57, 20.86, 20.63 ppm.

Synthesis of compound **5**. A mixture of **6** (1.26 g, 3.6 mmol), bis(pinacolato)diboron (3.65 g, 14.6 mmol), [1,1′-bis(diphenylphosphino)ferrocene]dichloropalladium(II) [Pd(dppf)Cl_2] (527 mg, 0.72 mmol), and potassium acetate (1.06 g, 10.77 mmol) in 1,4-dioxane (5 mL) was stirred under argon atmosphere at 100 °C for 24 h. After cooling, the product was washed with water and extracted with DCM (100 mL ×2). The obtained organic phase was dried with anhydrous $MgSO_4$ and then purified by silica gel column chromatography (DCM/petroleum ether 1:3 as eluent) to afford **5** (1.08g, 75%) as a white solid. Mass found: m/z 398.3 [M+1$^+$]; Calcd. for $C_{27}H_{31}BO_2$ m/z 397.245. Elementary analysis. Found: C, 81.32%, H, 7.63% Calcd. for $C_{27}H_{31}BO_2$: C, 81.41%, H, 7.84%.

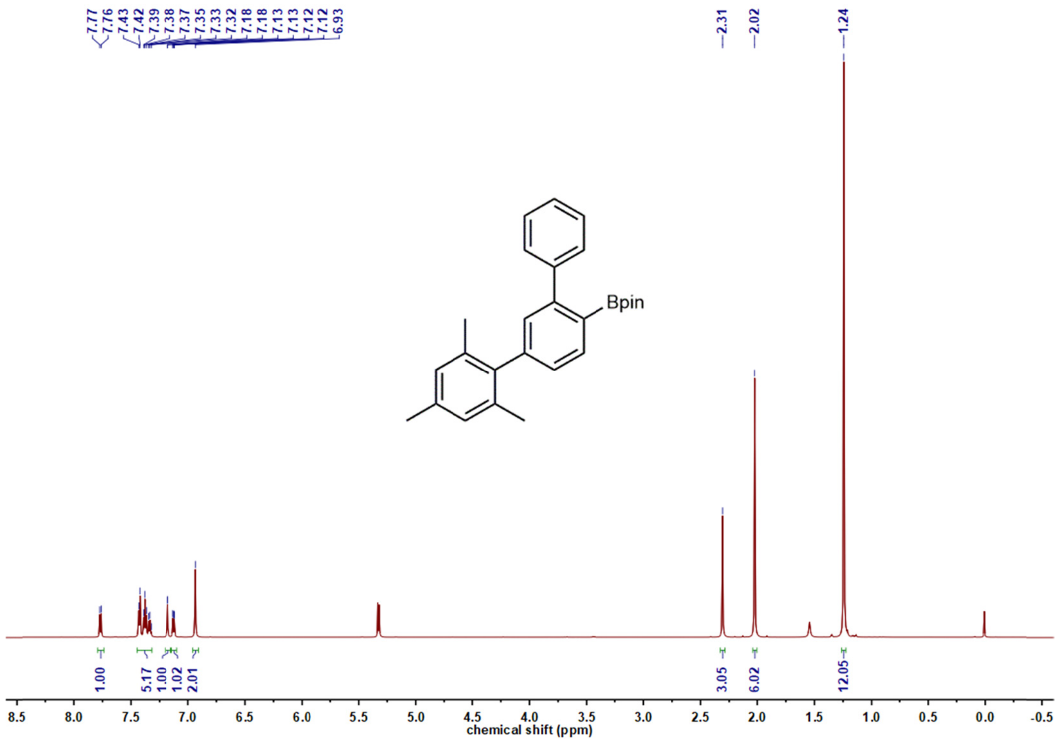

Supplementary Figure 35. ^{1}H NMR of compound 5. (600 MHz, CD_2Cl_2): δ = 7.77 (d, 1H), 7.43-7.32 (m, 5H), 7.18 (d, 1H), 7.12 (d, 1H), 6.93 (s, 2H), 2.31 (s, 3H), 2.02 (s, 6H), 1.24 (s, 12H) ppm.

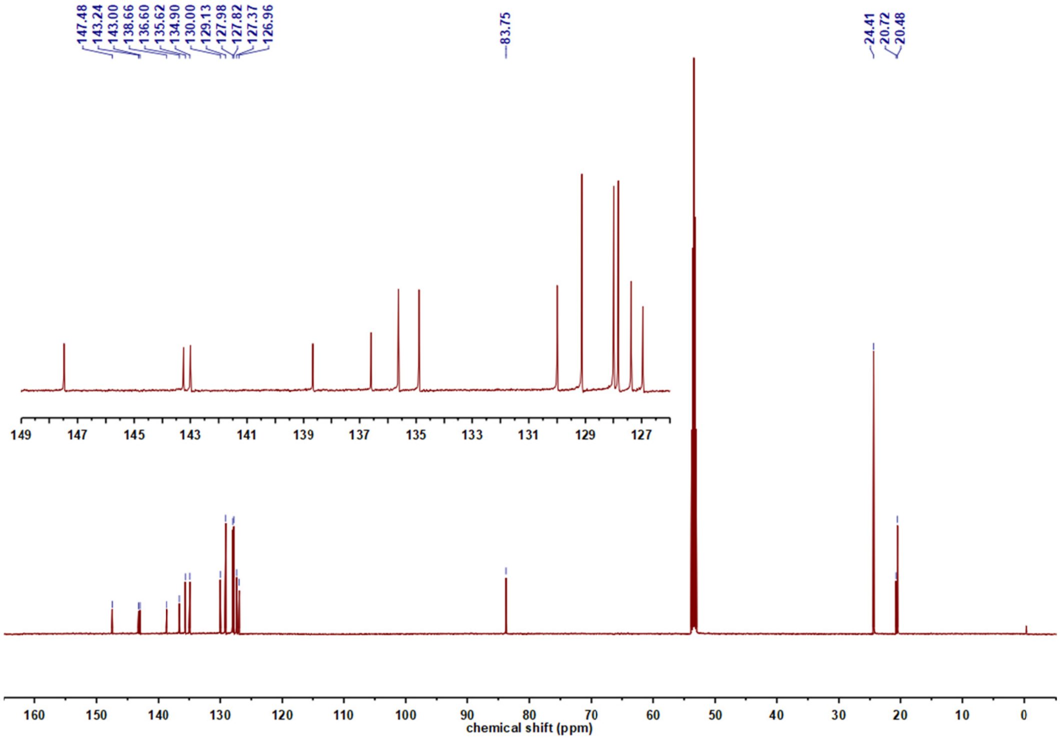

Supplementary Figure 36. ^{13}C NMR of compound 5. (150 MHz, CD_2Cl_2): δ = 147.48, 143.24, 143.00, 138.66, 136.60, 135.62, 134.90, 130.00, 129.13, 127.98, 127.82, 127.37, 126.96, 83.75, 24.41, 20.72, 20.48 ppm.

Synthesis of compound **4**. A mixture of bis(4-bromophenyl)acetylene (336 mg, 1 mmol), **5** (995 mg, 2.5 mmol), tris(dibenzylideneacetone)dipalladium(0) [$Pd_2(dba)_3$] (458 mg, 0.5 mmol), 2-dicyclohexylphosphino-2',6'-dimethoxybiphenyl (Sphos) (410 mg, 1.0 mmol), and Cs_2CO_3 (1.95 g, 6 mmol) in toluene (16 mL) and water (8 mL) was stirred under argon atmosphere at 100 °C for 24 h. After the reaction, the product was washed with water and extracted with DCM (50 mL × 2). The organic layer was dried with anhydrous $MgSO_4$ and concentrated under reduced pressure. The crude products were then purified by silica gel column chromatography (DCM/petroleum ether 1:4 as eluent) to afford **4** (403 mg, 56%) as a white solid. Mass found: m/z 718.4 [M^+]; Calcd. for $C_{56}H_{46}$ m/z 718.360. Elementary analysis. Found: C, 93.21%, H, 6.33% Calcd. for $C_{56}H_{46}$: C, 93.55 %, H, 6.45%.

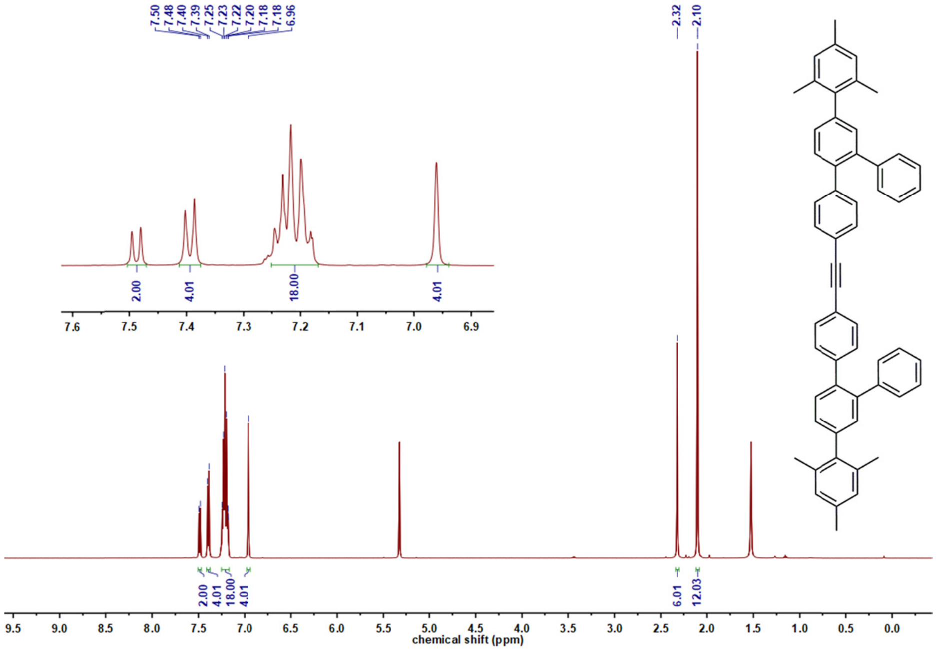

Supplementary Figure 37. ¹H NMR of compound 4. (600 MHz, CD_2Cl_2): δ = 7.49 (d, 2H), 7.39 (d, 4H), 7.25-7.18 (m, 18H), 6.96 (s, 4H), 2.32 (s, 6H), 2.10 (s, 12H) ppm.

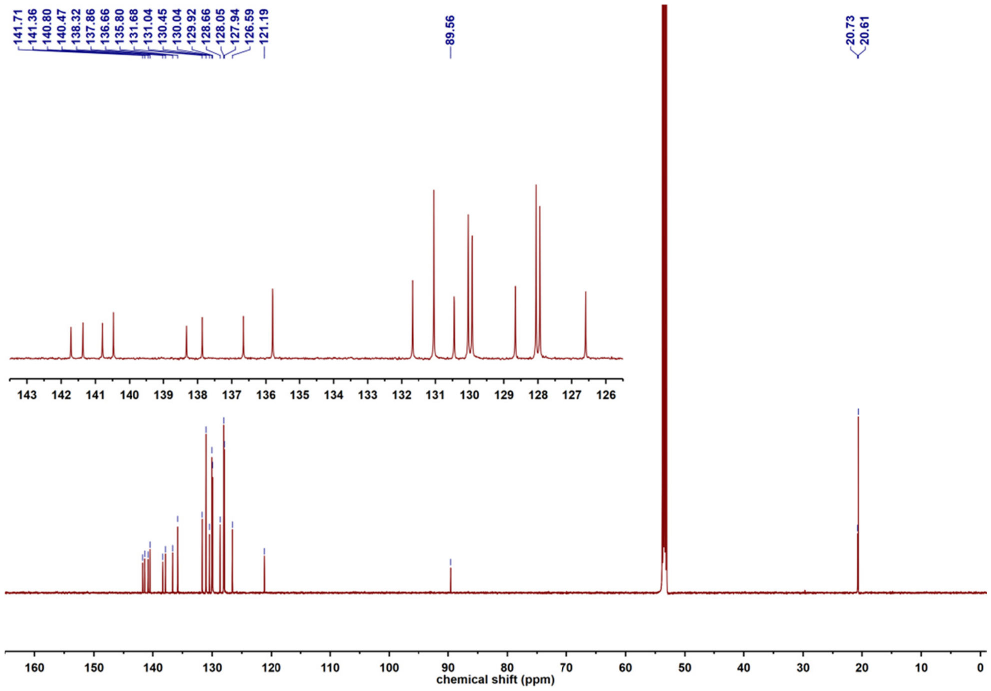

Supplementary Figure 38. ^{13}C NMR of compound 4. (150 MHz, CD_2Cl_2): δ = 141.71, 141.36, 140.80, 140.47, 138.32, 137.86, 136.66, 135.80, 131.68, 131.04, 130.45, 130.04, 129.92, 128.66, 128.05, 127.94, 126.59, 121.19, 89.56, 20.73, 20.61 ppm.

Synthesis of compound **3**. A mixture of **4** (80 mg, 0.11 mmol) and $Co_2(CO)_8$ (18.8 mg, 0.055 mmol) in anhydrous 1,4-dioxane (1.5 mL) was stirred overnight at reflux under argon atmosphere. After cooling, the mixture was washed with hydrochloric acid (1.0 mol L^{-1}) and extracted with DCM (10 mL × 2). The organic layer was washed with water, dried with anhydrous $MgSO_4$ and concentrated under reduced pressure. The crude products were further purified by silica gel column chromatography (DCM/petroleum ether = 1:3 as eluent) to afford **3** (36 mg, 45%) as a white solid. Mass found: m/z 2155.1 [M^+]; Calcd. for $C_{168}H_{138}$ m/z 2155.079.

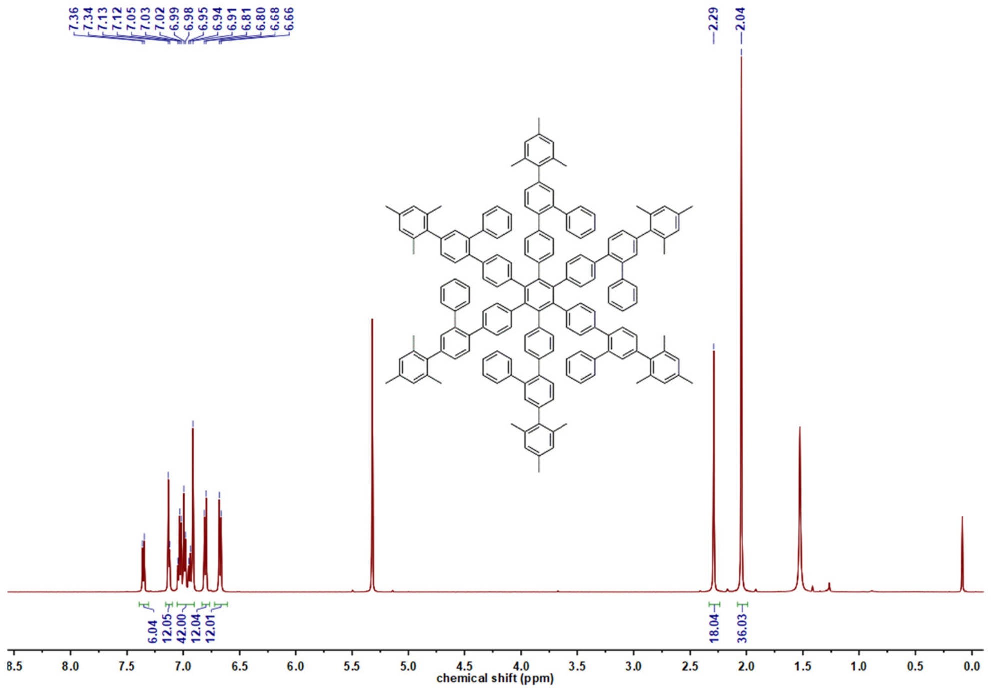

Supplementary Figure 39. ^{1}H NMR of compound 3. (600 MHz, CD_2Cl_2): δ = 7.35 (d, 6H), 7.13-7.12 (m, 12H), 7.05-6.91 (m, 42H), 6.80 (d, 12H), 6.67 (d, 12H), 2.29 (s, 18H), 2.04 (s, 36H) ppm.

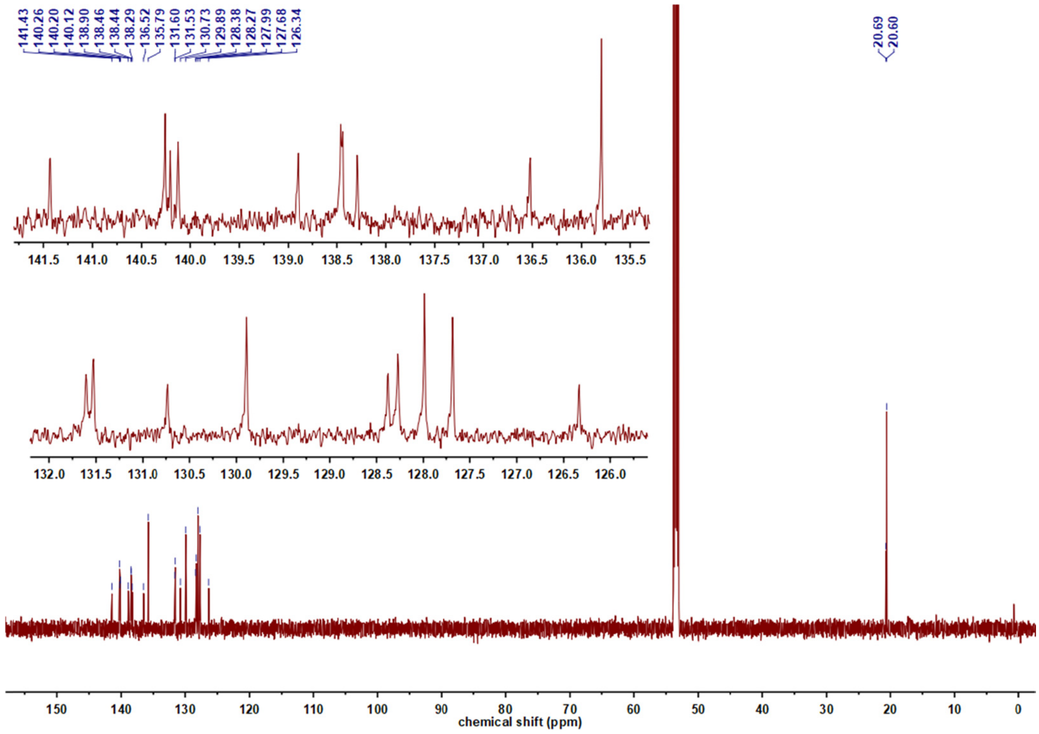

Supplementary Figure 40. ^{13}C NMR of compound 3. (150 MHz, CD_2Cl_2): δ = 141.43, 140.26, 140.20, 140.12, 138.90, 138.46, 138.44, 138.29, 136.52, 135.79, 131.60, 131.53, 130.73, 129.89, 128.38, 128.27, 127.99, 127.68, 126.34, 20.69, 20.60

ppm.

Synthesis of MBLG **1**. A solution of **3** (21 mg, 0.01 mmol) in DCM (20 mL) was degassed by argon bubbling for 10 min. Then iron(III) chloride (272 mg 1.68 mmol) was added. After stirring at room temperature for 1h under continuous bubbling with argon, the reaction was quenched by the addition of methanol. The crude products were further purified by silica gel column chromatography (DCM/petroleum ether = 1:1 as eluent) to afford **1** (2 mg, 10%) as a purple solid.

Synthesis of compound **9**.

3,4-bis(4-bromophenyl)-2,5-diphenyl-2,4-cyclopentadien-1-one (**10**) was prepared according to the reported procedure[1]. A mixture of **10** (2 g, 3.70 mmol), $Pd(PPh_3)_4$ (428 mg, 0.37 mmol), K_2CO_3 (1.02 g, 7.41 mmol), 2,4,6-trimethylphenylboronic acid (1.46 g, 8.84 mmol), in 1,4-dioxane (20 mL) and water (10 mL) was stirred under argon atmosphere at 80 ^oC for 24 h. After the reaction, the products were extracted with DCM (30 mL) and washed with water (20 mL). The combined organic layer was dried over $MgSO_4$ and concentrated in vacuum. The crude mixture was purified by silica gel column chromatography (ethyl acetate/petroleum ether = 4:1 as eluent), and the compound **9** was obtained as a dark red solid with a yield of 85% (1.95 g, 3.15 mmol). Mass found: m/z 620.3 [M+]; Calcd. for $C_{47}H_{40}O$ m/z 620.307. Elementary analysis. Found: C, 90.71%, H, 6.34% Calcd. for $C_{47}H_{40}O$: C, 90.93 %, H, 6.49%.

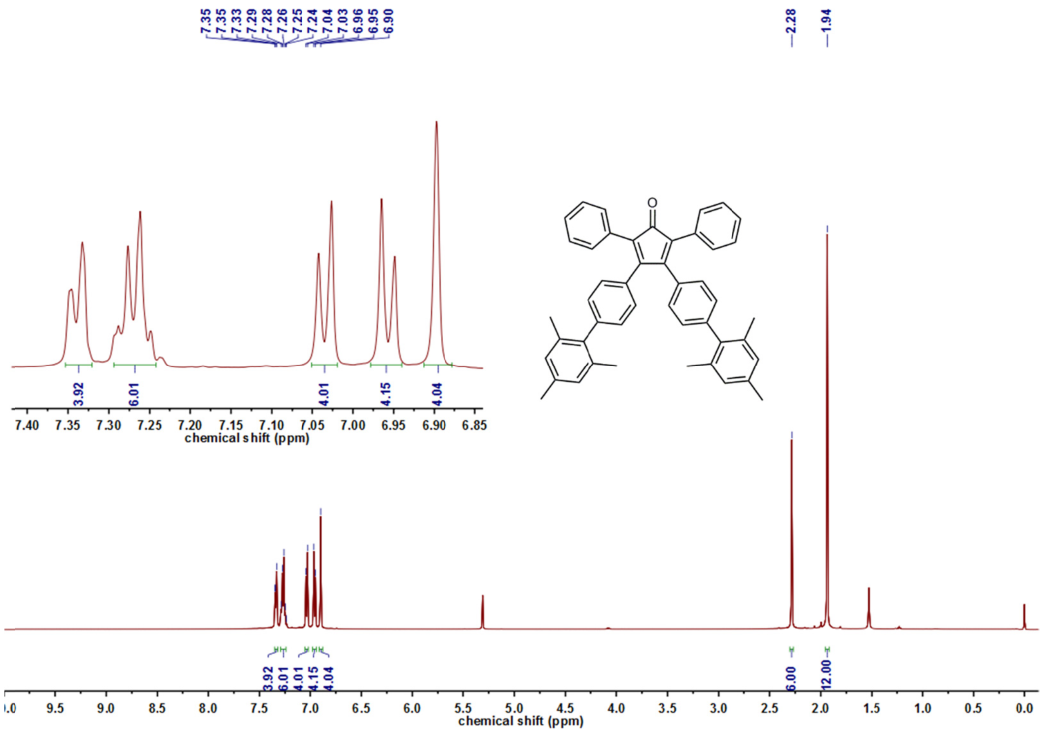

Supplementary Figure 41. ^{1}H NMR of compound 9. (500 MHz, CD_2Cl_2): δ = 7.35 (d, 4H), 7.29-7.24 (m, 6H), 7.03 (d, 4H,), 6.95 (d, 4H), 6.90 (s, 4H), 2.28 (s, 6H), 1.94 (2, 12H) ppm.

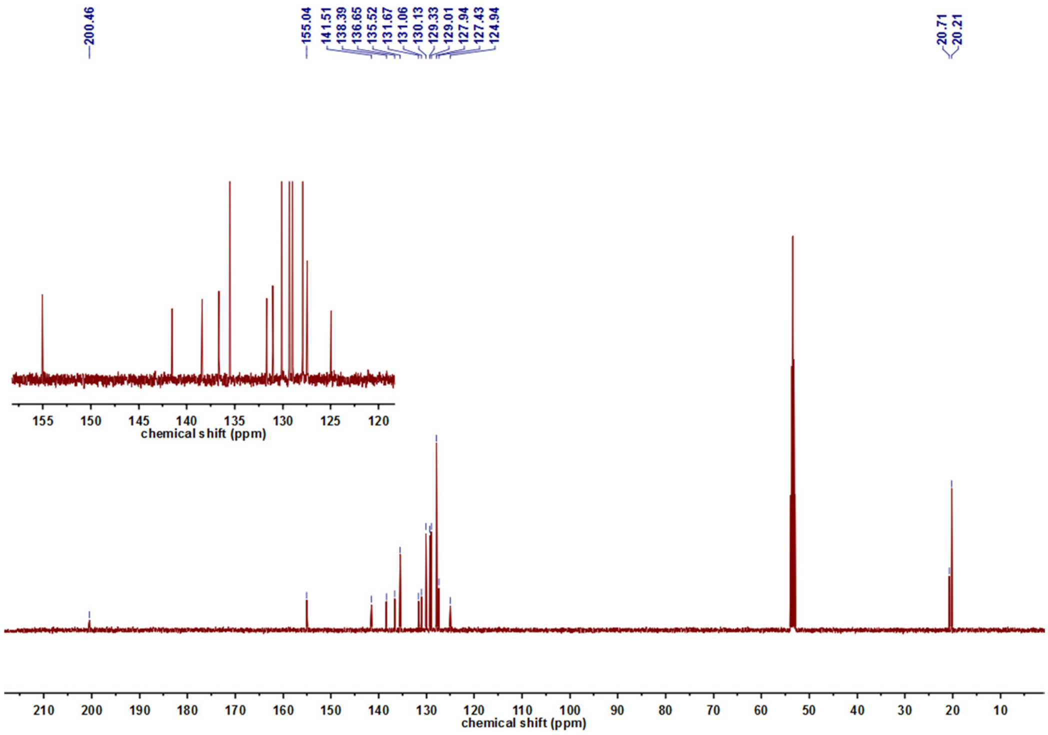

Supplementary Figure 42. ^{13}C NMR of compound 9. (125 MHz, CD_2Cl_2) δ = 200.46, 155.04, 141.51, 138.39, 136.65, 135.52, 131.67, 131.06, 130.13, 129.33, 129.01, 127.94, 127.43, 124.94, 20.71, 20.21 ppm.

Synthesis of compound **8**

A mixture of **9** (1.2 g, 2 mmol) and 1,3,5-triethynylbenzene (91 mg, 0.61 mmol) in diphenyl ether (4 mL) was refluxed for 12 h. After cooling down to room temperature, methanol was added. The precipitate was collected by filtration and then purified by silica gel column chromatography (DCM/petroleum ether 1:3 as eluent). The compound **8** was obtained as a white solid and the yield of **8** is about 45%. The mass measurement of **8** is failed due to its low ionization efficiency, using the MALDI or APCI (atmospheric-pressure chemical ionization) or ESI (electrospray ionization) source.

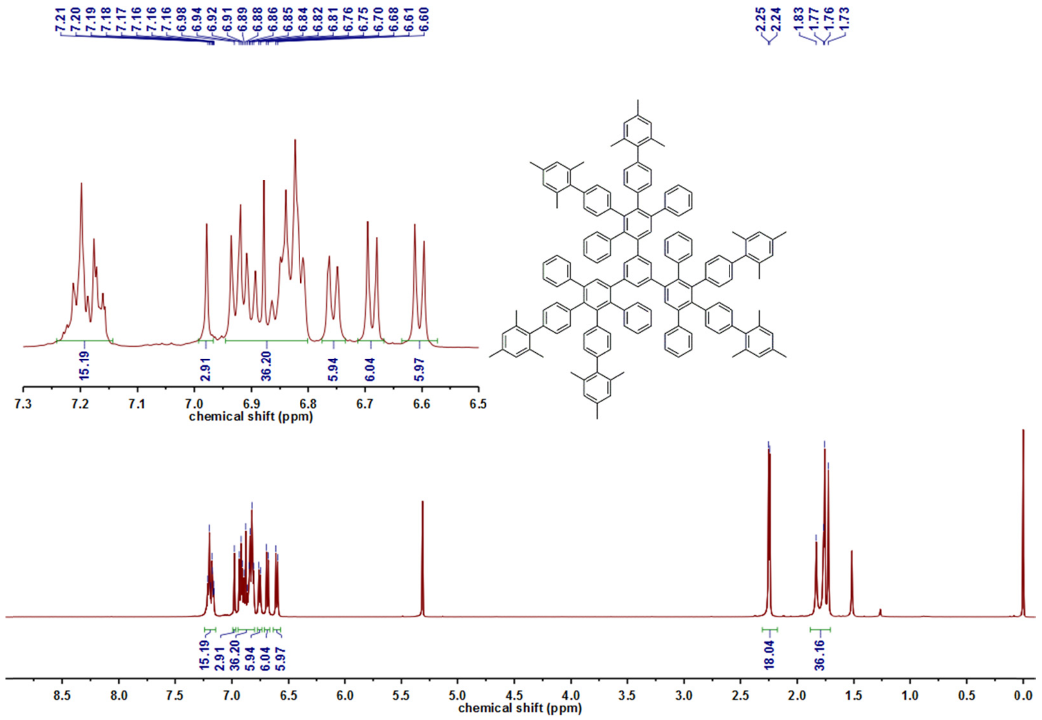

Supplementary Figure 43. ^{1}H NMR of compound 8. (600 MHz, CD_2Cl_2) δ = 7.27-7.21 (m, 15H), 7.03 (s, 3H), 6.98-6.86 (m, 36H), 6.78-6.74 (d, 6H), 6.71-6.67 (d, 6H), 6.64-6.57 (d, 6H), 2.31-2.17 (d, 18H), 1.88-1.70 (m, 36H) ppm.

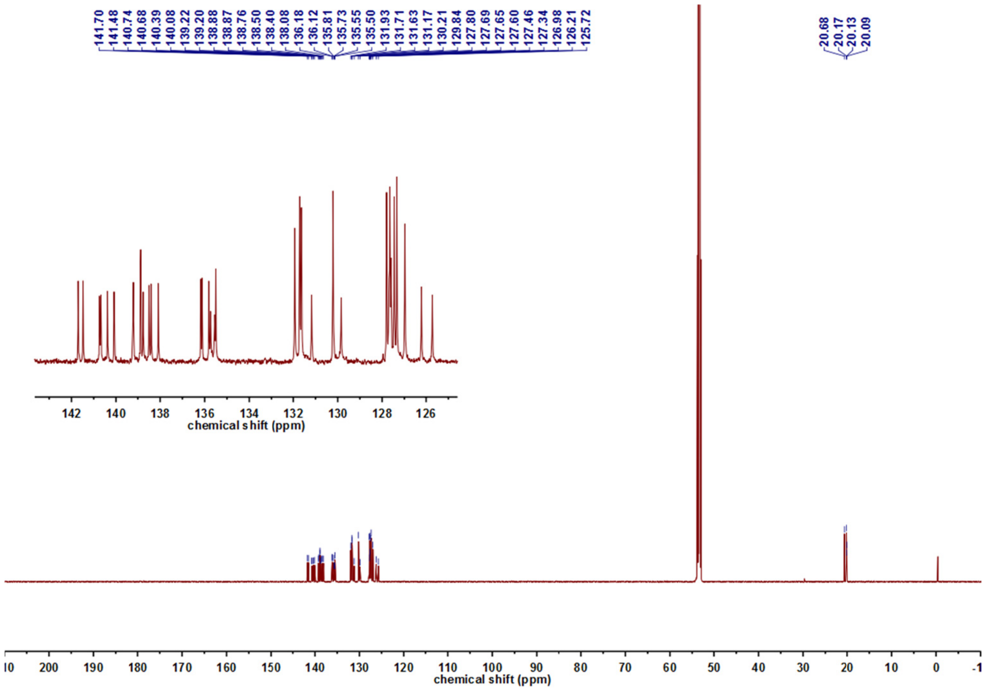

Supplementary Figure 44. ^{13}C NMR of compound 8. (150 MHz, CD_2Cl_2) δ = 141.70, 141.48, 140.74, 140.68, 140.39, 140.08, 139.22, 139.20, 138.88, 138.87, 138.76, 138.50, 138.40, 138.08, 136.18, 136.12, 135.81, 135.73, 135.55, 135.50, 131.93, 131.71, 131.63, 131.17, 130.21, 129.84, 127.80, 127.69, 127.65, 127.60, 127.46, 127.34, 126.98, 126.21, 125.72, 20.68, 20.17, 20.13, 20.09 ppm.

Synthesis of MBLG 2

A solution of **8** (100 mg, 0.0519 mmol) in DCM (100 mL) was degassed by argon bubbling for 10 min. Then iron(III) chloride (908 mg, 5.61 mmol) was added. The reactants were stirring under continuous bubbling with argon at room temperature for 2 h and then quenched by methanol. Then the red precipitate formed and was filtrated. The precipitate was purified by silica gel column chromatography (DCM/petroleum ether = 1:2 as eluent) and MBLG **2** was obtained as a red solid and the yield of **2** is about 52% (51 mg).

Supplementary Note 2

NMR assignment for 1

The peaks at 10.13 and 9.96 ppm are assigned to the protons at the nanographene core of **1**, due to their much larger chemical shift compared with the hydrogen at the benzene rings. The number of peaks for the protons at the C_{114} core is consistent with the C_{6v} molecular symmetry of **1**. The peaks at 7.82 and 7.33 ppm are assigned to the protons at the benzene ring of peripheral mesityl group, the chemical shifts of which agree with those of previously reported compounds with mesityl substituents. The peaks at 4.05, 2.86 and 2.06 ppm are assigned to the protons of methyl groups of mesityl groups. The intensity ratio of all peaks matches well the structure of **1**.

The exclusive assignment of each peaks to each proton species in **1** is achieved with the assistance of NOESY. From the NOESY correlation between protons at the C_{114} core and those at methyl groups (Supplementary Figure 22), the peak at 4.05 ppm is assigned to the inward-facing methyl group (H_g), because it has much stronger coupling with the protons at the C_{114} core (H_a and H_b). Therefore, the peak at 2.06 ppm is assigned to the outward-facing methyl group (H_c).

The protons (H_a and H_b) at the inner core have different distances to the peripheral mesityl, and H_b is relatively closer to mesityl group than H_a. The coupling difference between protons at the inner core and protons of outward-spacing methyl groups could reflect the difference in distance more clearly, because only the intralayer spatial H···H couplings exist between them (Supplementary Figure 10). As shown in Supplementary Figure 22, the peak at 9.96 ppm reveals a larger NOE signal with the outward-spacing methyl group (H_c) and thus is assigned to H_b. Then, the peak at 10.13 ppm is assigned to H_a.

Using the NOESY correlation between the aromatic (H_f and H_d) and methyl (H_c, H_g and H_e) protons of mesityl group, we can assign the peak at 2.86 ppm to the para methyl group (H_e), because only the para methyl group is sufficient close to both aromatic protons (H_f and H_d) (Supplementary Figure 23). The inward-facing aromatic proton of mesityl (H_f) is in short distance with inward-facing (H_g) and para methyl

(H_e) groups (Supplementary Figure 23), and thus we can assign the peak at 7.82 ppm to the inward-facing aromatic proton of mesityl group (H_f). Also, the peak at 7.33 ppm can be assigned to the outward-facing aromatic proton of mesityl group (H_d). The experimental assignment of all the hydrogens of **2** fits well the theoretical NMR spectrum computed with the structure of **2**.

NMR assignment for 2

Similarly, the NMR peaks of **2** can be classified into three categories, i.e. protons at the inner nanographene core (11.13-8.17 ppm), those at the benzene ring of mesityl group (7.55-7.18 ppm) and at the methyl groups (3.39-1.98 ppm) (Supplementary Figure 24). The number of peaks for the protons at the inner C_{96} core is consistent with its C_3 molecular symmetry. The intensity ratio of all peaks matches well the structure of **2**.

First, the H_j can be easily and exclusively assigned, because it is the sole triplet in the ^1H NMR spectrum (Supplementary Figure 24). Then the two doublet (9.24 and 9.11 ppm) should be H_i and H_k, which is also confirmed by the ^1H-^1H COSY spectrum (Supplementary Figure 24).

To distinguish H_k and H_i, we check the NOE signals among the protons at the C_{96} core (Supplementary Figure 26). As shown in asymmetric unit of **2** (insert figure in Supplementary Figure 26), H_k is in short distance with three protons (H_j, H_l and H_m) at the C_{96} core, whereas H_i is close to two protons (H_j and H_h). Indeed, the doublet peak at 9.24 ppm correlates with three proton signals and therefore is assigned to H_k (Supplementary Figure 26). On the other hand, the doublet peak at 9.11 ppm correlates to two proton signals and is assigned to H_i (Supplementary Figure 26). Also, H_h can be assigned to the peak at 9.18 ppm due to its correlation with H_i. The peaks at 11.13 and 10.26 should be H_l and H_m. The rest two peaks at 9.73 and 9.38 ppm should be H_s and H_t (Supplementary Figure 26).

According the C_3 symmetry of C_{96} core, there are two inequivalent mesityl groups (A and B) (Supplementary Figure 27). Indeed, based on the NOESY correlations between the aromatic and methyl protons of mesityl groups (Supplementary Figure 27), the peaks at 7.55, 7.18, 3.41, 2.63 and 1.99 ppm are grouped into one mesityl

group and the peaks of 7.44, 7.42, 3.26 2.92 and 2.31 ppm belong to the other.

As shown in Supplementary Figure 28, mesityl A is close to H_h whereas mesityl B is far away. So, methyl groups (H_u and H_y) of mesityl A are close to H_h and their spatial coupling could be seen in NOESY (highlighted by the dashed blue line in Supplementary Figure 28). Therefore, the peaks at 3.41 and 1.99 ppm should come from H_y and H_u. The much stronger NOE of peak at 3.41 ppm to H_h compared with that of peak at 1.99 ppm is ascribed to the interlayer proton coupling due to the bilayer structure, which can help identify the signal at 3.41 ppm as the inward-spacing methyl group (H_y) of mesityl A and the signal at 1.99 ppm as the outward-spacing methyl group (H_u). Once H_y and H_u are assigned, the rest signals of mesityl A could be identified based on Supplementary Figure 27.

The last signal group (7.44, 7.42, 3.26 2.92 and 2.31 ppm) come from mesityl B. The stronger coupling between the signal at 3.26 ppm and protons at the C_{96} core (H_m, H_l and H_j) (Supplementary Figure 28) indicates the peak at 3.26 ppm should be the inward-spacing methyl group (H_n). Consequently, H_r, H_o, H_q and H_p correspond to the peaks at 2.31, 7.44, 7.42 and 2.92 ppm, respectively.

After the assignment of all the signals for peripheral mesityl groups, the exclusive assignment between H_l and H_m and between H_s and H_t is possible based on their different distance to peripheral mesityl groups. The coupling difference between protons at the inner core and protons of outward-spacing methyl groups could reflect the difference in distance more clearly, because only the intralayer spatial H···H couplings exist between them (Figure 3A). H_m is closer to outward-spacing methyl (H_r) at the mesityl B, compared to H_l, so the NOE signal between H_m and H_r should be stronger. As shown in Supplementary Figure 28, the correlation of the peak at 10.26 ppm to the signal of H_r (2.31 ppm) is observed (highlighted by the dashed orange cycle in Supplementary Figure 28) and that between the peak at 11.13 ppm to H_r is not shown. Then, we could assign the peak at 10.26 ppm to H_m and 11.13 ppm to H_l. Similarly, we could identify H_s and H_t correspond to the peaks at 9.73 and 9.38 ppm, respectively.

We could see in Supplementary Figure 29, all the inward-spacing methyl groups

(H_y and H_u) have obviously stronger coupling to the protons at inner C_{96} core, compared to that of outward-spacing methyl groups (H_r and H_u), which clearly validates the bilayer structure of **2**.

As shown in Supplementary Figures 30-32, the NOE correlations found in **2'** are in good agreement with those found in **2**, which indicates **2** and **2'** have the same bilayer structure. The proton couplings through space found by NOESY is consistent with those found in the crystal structure of **2'**. With the help of NOESY, all the peaks of **2'** are assigned, as the assignment for **2**.

Supplementary Note 3

Computational setup and models of MBLGs

Density functional theory (DFT) calculations were carried out using the freely available program package CP2K/Quickstep.[2] The DFT implementation in Quickstep is based on a hybrid Gaussian plane wave (GPW) scheme. Orbitals are described by an atom-centered Gaussian-type basis set, while an auxiliary plane wave basis is used to re-expand the electron density.[3] The wave function optimization is performed using an efficient orbital transformation minimizer, which avoids the traditional matrix diagonalization method and gives optimal convergence control.[4] Analytic Goedecker-Teter-Hutter (GTH) pseudopotentials[5] were employed to represent the core electrons. The basis sets for the valence electrons ($1s^1$ for H, $2s^2 2p^2$ for C and $3s^2$ $3p^4$ for S) consist of short-ranged (less diffuse) double-ζ basis functions with one set of polarization functions (DZVP).[6] The gradient-corrected Perdew–Burke–Ernzerhof (GGA-PBE) functional[7] was used for all calculations with the Grimme's dispersion correction.[8] The plane wave basis for the electron density expansion is cut off at 400 Ry. All the simulations only used the Γ point of the supercell for expansion of the orbitals considering the large size of the cell. The convergence criterion for wave function optimization was set by a maximum electronic gradient of 3×10^{-7} a.u. and an energy difference tolerance between self-consistent field (SCF) cycles of 10^{-13} a.u.

The self-consistent continuum solvation (SCCS)[7, 9-11] model was used to account for the solvation effect. In this approach, a dielectric cavity is constructed at the surface of the system. The dielectric function is defined as:

$$\varepsilon = \exp[(\frac{\ln\rho_{max} - \ln\rho}{\ln\rho_{max} - \ln\rho_{min}} - \sin(2\pi \frac{\ln\rho_{max} - \ln\rho}{\ln\rho_{max} - \ln\rho_{min}}) / 2\pi)\ \ln\varepsilon_0] \quad (1)$$

where ε_0 is the dielectric constant of the bulk solvent, the ρ_{max} and ρ_{min} are the density thresholds that delimit the internal and external iso-contours of the smooth dielectric cavity. Non-electrostatic effects such as the cavitation energy, dispersion and repulsion effects are also included in this model. These contributions are expressed as $G_{cav} = \gamma S$ and $G_{dis+rep} = \alpha S + \beta V$, where γ is the experimental solvent surface tension,

α and β are fitted parameters, S is the quantum surface and V is the solute volume. The experimental dielectric constant $\varepsilon_0 = 2.641$ and solvent surface tension $\gamma = 32.3$ dyn cm^{-1} of CS_2 were used in our calculations. In specific terms, we employ the following parameterization of the solvent, which has been extensively fitted on more than 240 molecules and found to be in good agreement with both experiment and the widely used polarizable continuum model (PCM) as implemented in Gaussian03[10]: $\rho_{min} = 10^{-4}$ a.u., $\rho_{max} = 5\times10^{-3}$ a.u., α = -20.8 dyn cm^{-1}, and β = 0 GPa.

For MBLGs, very large supercells with the sizes of up to 40 × 40 × 40 $Å^3$ were used. The initial structures were obtained from experiment, and then optimized with DFT. Full 3D periodic boundary conditions (PBC) were applied. When calculating the energies of the CS_2 molecule in gas phase and liquid phase, we first performed DFT based molecular dynamics (DFTMD) simulations. The time step for the MD runs is 0.5 fs. Canonical ensemble conditions were imposed by a Nosé -Hoover thermostat with a target temperature of 330 K for gas phase CS_2 and 298K for liquid phase CS_2. About 5 ps DFTMD runs were performed to obtain the stable structures, which were then used as initial structures for static geometry optimization using the BFGS minimizer.

Calculation of binding energies

To determine the stability of the MBLGs, we calculated the binding energies of the MBLGs in solvent CS_2. The method of the binding energy calculation in solvent is introduced below.

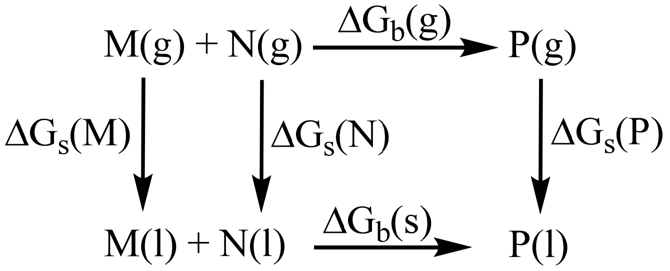

Supplementary Figure 45. Thermodynamic cycle for the binding reaction.

For an association reaction $M(l) + N(l) \rightarrow P(l)$, the binding free energy was calculated by using the thermodynamic cycle shown in Supplementary Figure 45.

Here, $\Delta G_b(g)$ and $\Delta G_b(s)$ are the binding free energies in gas phase and liquid solution, respectively. $\Delta G_s(M)$, $\Delta G_s(N)$ and $\Delta G_s(P)$ are the solvation free energies of M, N and P, respectively. From this cycle, the $\Delta G_b(s)$ can be expressed as,

$$\Delta G_b(s) = \Delta G_b(g) + \Delta G_s(P) - \Delta G_s(M) - \Delta G_s(N) \quad (2)$$

In our calculation, the total energy difference $\Delta E_b(g)$ is used to estimate the free energy change $\Delta G_b(g)$ assuming the entropic contributions are small. Thus, the expression for the binding energy in our calculation is given by

$$\Delta G_b(s) = \Delta E_b(g) + \Delta G_s(P) - \Delta G_s(M) - \Delta G_s(N) \quad (3)$$

Solvation energies were obtained using a mixed implicit-explicit solvation approach, which is illustrated in Supplementary Figure 46.

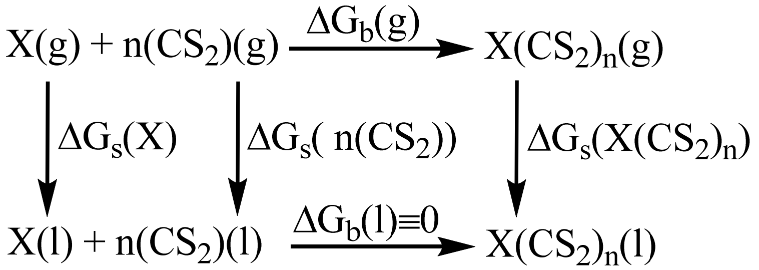

Supplementary Figure 46. Thermodynamic cycle to compute $\Delta G_s(X)$ within a mixed implicit-explicit solvation approach.

The calculated binding energies were listed in Supplementary Table 1. As can be seen, the binding energies of MBLGs are large, which means the formation of the bilayer structure are thermodynamically favorable, which is consistent with the experimentally observed stability of the MBLGs. Note that we have also calculated the binding structures and energies using an implicit solvation model, which have considerable differences from those obtained using the mixed implicit-explicit solvation approach, indicating that the interaction between explicit CS_2 and MBLGs is significant and cannot be neglected.

There is however an issue that our calculated binding energies for the trimer

formation are moderately negative, which seems to suggest oligomerization would be still favored over dimerization. This could be explained by two accounts. Firstly, DFT (with dispersion corrections) tends to overestimate the binding energies of aromatic systems. For example, Mackie and DiLabio calculated the aggregation of the hexa-n-hexyl-hexa-peri-hexabenzocoronene using DFT with dispersion corrections[12], and obtained a binding energy for dimer formation of -2.27 eV. While, disaggregation of the dimer structure can be observed experimentally at low concentration[13,14]. We have also tested on the dimerization energy of tri-mesityl hexa-peri-hexabenzocoronene (the structure is shown below). Although the molecule doesn't aggregate from experiment, DFT calculation still gives a binding energy of -1.63 eV. We have also calculated the binding energy of for dimer formation of MBLG **1** using more advanced vdW correction method, namely, many-body dispersion with range-separated self-consistent screening (MBD@rsSCS)[15-17], in comparison with the Grimme's dispersion method. We have found that the optimized geometry of stacked MBLG 1 with the MBD@rsSCS method is almost the same as that with the Grimme's correction method, and there is only a very small difference of in the binding energy.

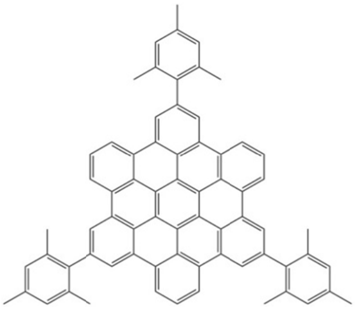

Supplementary Figure 47. Structure of tri-mesityl hexa-peri-hexabenzocoronene.

Secondly, the present solvation model may not accurately account for all important contributions to solvation free energies, i.e. entropic terms. Addition of some explicit CS_2 solvent molecules in a mixed implicit-explicit solvation approach, can considerably change the binding energies, compared to the implicit solvation model widely used in literature. However, this approach might still not be sufficient, and lead to overestimated binding energies. Going beyond static solvation models, one would

have to carry out molecular dynamics and free energy calculation (i.e. potential of mean force) on fully atomistic models (both solute and solvent molecules), which is however extremely expensive at the level of DFT. It may merit future study, but is beyond the scope of the present work.

Calculation of NMR spectra

The ^{1}H-NMR spectra of MBLGs (orange lines in Figure 2d and 2h) and monolayer of MBLG 1 (Supplementary Figure 4) were calculated for comparison with NMR experiment to help resolve the structures of MBLGs. These calculations were performed using the GAUSSIAN 09 program package.[18] The B3LYP functional[19-21] and the 6-31G* basis sets were employed. The calculated ^{1}H-NMR spectra of MBLGs (orange lines in Figure 2d and 2h) are in good agreement with the experimental patterns, confirming the bilayer structures and other geometric features of MBLGs.

Supplementary Note 4

Comparison of optical properties of 2.

The emission spectra of aromatic compounds depend on the molecular structure, environment and aggregation state, and direct comparison between the monomer and dimer in the same dispersion is the ideal case. However, we took effort to dissociate MBLGs into monomers by varying temperature and solvents, but failed due to the high stability of MBLGs. Thus, direct comparison between MBLG with its monomer cannot be achieved at the current stage.

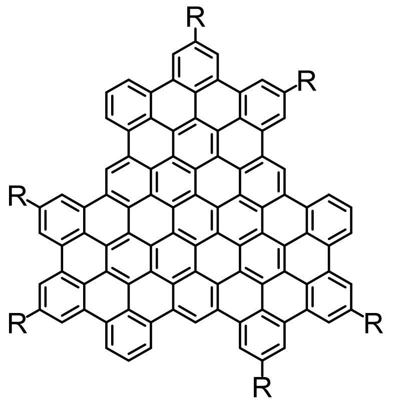

Supplementary Figure 48. The molecular structure of $C_{96}H_{24}(C_{12}H_{25})_6$ (R=n-$C_{12}H_{25}$) and monomer of 2 (R=mesityl)

A monomer PL spectrum of an analogous molecule, $C_{96}H_{24}(C_{12}H_{25})_6$, was obtained from the single molecule technique[22]. Compared with the monomer of **2**, this molecule has the same aromatic core and molecular symmetry, but different peripheral groups. As the electronic structures of molecules are primarily determined by the aromatic core and symmetry, we can expect the similar spectral shape between these two monomers. Therefore, the single molecule PL spectrum of $C_{96}H_{24}(C_{12}H_{25})_6$ monomer reported[22] is used as a prototype to interpret the spectral modification arising from dimerization.

Unfortunately, we are not able to use the PL spectrum of $C_{96}H_{24}(C_{12}H_{25})_6$ monomer in solution as the reference because $C_{96}H_{24}(C_{12}H_{25})_6$ ensemble in solution consists of monomers and other aggregates[23]. The monomer PL spectrum however could be obtained in Ref. 22 because the monomers and aggregates were immobilized in the PS

matrix and the single molecule PL technique could select the monomers out of the aggregates. However, the PL quantum yield could be varied due to the other factors rather than dimer formation. A solution with pure monomers cannot be obtained at current stage. To avoid confusion, we prefer not to directly compare the quantum yield with monomer trapped in matrix.

Supplementary Note 5

Transient absorption (TA) spectroscopic measurement

The TA measurement is based on the Ti:sapphire ultrafast laser amplifier (Coherent Astrella, 800 nm, pulse duration ~100 fs, ~6 mJ/pulse and 1 KHz repetition rate) and the pump-probe transient absorption spectrometer (Eos Fire, Ultrafast System). Half of the output power of the amplifier is delivered to the pump-probe setup. The pump pulse (400 nm) is generated by doubling the frequency of the fundamental pulse (800 nm) based on a BBO crystal, and its intensity is attenuated by two neutral density filter wheels. The broadband probe pulse (350-900 nm) is provided by a sub-nanosecond photonic crystal fiber based supercontinuum laser. Thus, the time resolution of the transient absorption spectrometer is determined by the probe pulse duration (~1 ns). The pump-probe delay is controlled electronically, and the maximum time window is set up to 20 microsecond. The pump and probe are focused and overlapped into a cuvette containing the sample solution.

Supplementary Table

**Binding Energy (eV)**	**1L+1L→2L**	**1L+2L→3L**
**MBLG 1**	**-4.58**	**-3.31**
**MBLG 2**	**-2.98**	**-2.09**

Supplementary Table 1. Binding energies (in eV) in solvent CS_2 by using the mixed implicit-explicit solvation model and the DFT-PBE functional.

Supplementary references

1. Huang, Y. et al. Temperature-dependent multidimensional self-assembly of polyphenylene-based “rod–coil” graft polymers. *J. Am. Chem. Soc.* **137**, 11602-11605 (2015).
2. VandeVondele, J. et al. Quickstep: Fast and accurate density functional calculations using a mixed Gaussian and plane waves approach. *Comput. Phys. Commun.* **167**, 103-128 (2005).
3. Lippert, B.G., Parrinello, J.H. & Michele A hybrid Gaussian and plane wave density functional scheme. *Mole. Phys.* **92**, 477-488 (1997).
4. VandeVondele, J. & Hutter, J. An efficient orbital transformation method for electronic structure calculations. *J. Chem. Phys.* **118**, 4365-4369 (2003).
5. Hartwigsen, C., Goedecker, S. & Hutter, J. Relativistic separable dual-space Gaussian pseudopotentials from H to Rn. *Phys. Rev. B* **58**, 3641-3662 (1998).
6. VandeVondele, J. & Hutter, J. Gaussian basis sets for accurate calculations on molecular systems in gas and condensed phases. *J. Chem. Phys.* **127**, 114105 (2007).
7. Scherlis, D.A., Fattebert, J.-L., Gygi, F., Cococcioni, M. & Marzari, N. A unified electrostatic and cavitation model for first-principles molecular dynamics in solution. *J. Chem. Phys.* **124**, 074103 (2006).
8. Grimme, S., Antony, J., Ehrlich, S. & Krieg, H. A consistent and accurate ab initio parametrization of density functional dispersion correction (DFT-D) for the 94 elements H-Pu. *J. Chem. Phys.* **132**, 154104 (2010).
9. Andreussi, O., Dabo, I. & Marzari, N. Revised self-consistent continuum solvation in electronic-structure calculations. *J. Chem. Phys.* **136**, 064102 (2012).
10. Fattebert, J.-L. & Gygi, F. Density functional theory for efficient ab initio molecular dynamics simulations in solution. *J. Comput. Chem.* **23**, 662-666 (2002).
11. Keilbart, N., Okada, Y., Feehan, A., Higai, S.i. & Dabo, I. Quantum-continuum simulation of the electrochemical response of pseudocapacitor electrodes under realistic conditions. *Phys. Rev. B* **95**, 115423 (2017).
12. Mackie, I.D. & DiLabio, G.A. Interactions in large, polyaromatic hydrocarbon dimers: application of density functional theory with dispersion corrections. *J. Phys. Chem. A* **112**, 10968-10976 (2008).
13. Chebny, V.J., Gwengo, C., Gardinier, J.R. & Rathore, R. Synthesis and electronic properties of iso-alkyl substituted hexa-peri-hexabenzocoronenes (HBC’s) from a versatile new HBC synthon, hexakis(4-acetylphenyl)benzene. *Tetrahedron Lett.* **49**, 4869-4872 (2008).
14. Wadumethrige, S.H. & Rathore, R. A facile synthesis of elusive alkoxy-substituted hexa-peri-hexabenzocoronene. *Org. Lett.* **10**, 5139-5142 (2008).
15. Hermann, J., Alfè, D. & Tkatchenko, A. Nanoscale π–π stacked molecules are bound by collective charge fluctuations. *Nat. Commun.* **8**, 14052 (2017).
16. Hermann, J., DiStasio, R.A. & Tkatchenko, A. First-principles models for van der Waals Interactions in molecules and materials: concepts, theory, and applications. *Chem. Rev.* **117**, 4714-4758 (2017).
17. Björkman, T., Gulans, A., Krasheninnikov, A.V. & Nieminen, R.M. van der Waals bonding in layered compounds from advanced density-functional first-principles calculations. *Phys. Rev. Lett.* **108**, 235502 (2012).
18. Frisch, M. J. et al. Gaussian 09 Revision D.01 (Gaussian Inc. Wallingford CT, 2009).

19. Becke, A.D. Density-functional exchange-energy approximation with correct asymptotic behavior. *Phys. Rev. A* **38**, 3098-3100 (1988).
20. Lee, C., Yang, W. & Parr, R.G. Development of the Colle-Salvetti correlation-energy formula into a functional of the electron density. *Phys. Rev. B* **37**, 785-789 (1988).
21. Becke, A.D. Density-functional thermochemistry. III. The role of exact exchange. *J. Chem. Phys.* **98**, 5648-5652 (1993).
22. Zhao, S. et al. Single photon emission from graphene quantum dots at room temperature. *Nat. Commun.* **9**, 3470 (2018).
